# Moral Injury: How It Affects Us and Tools to Combat It

**DOI:** 10.15766/mep_2374-8265.11357

**Published:** 2023-11-03

**Authors:** Connor Arquette, Valerie Peicher, Antonette Ajayi, Dora Alvarez, Alice Mao, Tram Nguyen, Anthony Sawyer, Connie Martin Sears, Eugene J. Carragee, Baraka Floyd, Bernadett Mahanay, Rebecca Blankenburg

**Affiliations:** 1 Fourth-Year Resident, Department of Plastic Surgery, Stanford University School of Medicine; 2 Postdoctoral Fellow, Department of Pediatrics, Stanford University School of Medicine; 3 Fellow, Department of Pediatrics, Stanford University School of Medicine; 4 Third-Year Resident, Department of Pediatrics, Stanford University School of Medicine; 5 Fellow, Department of Medicine, Stanford University School of Medicine; 6 Fourth-Year Resident, Department of Anesthesia, Stanford University School of Medicine; 7 Fourth-Year Resident, Department of Ophthalmology, Stanford University School of Medicine; 8 Professor, Department of Orthopedic Surgery, Stanford University School of Medicine; 9 Assistant Professor, Department of Pediatrics, Stanford University School of Medicine; 10 Fellowship Programs Manager, Department of Anesthesia, Stanford University School of Medicine; 11 Professor, Department of Pediatrics, Stanford University School of Medicine

**Keywords:** Burnout, Moral Injury, Case-Based Learning, Flipped Classroom, Diversity, Equity, Inclusion

## Abstract

**Introduction:**

Moral injury comprises feelings of guilt, despair, shame, and/or helplessness from having one's morals transgressed. Those underrepresented in health care are more likely to experience moral injury arising from micro- and macroaggressions. This workshop was designed for interprofessional health care providers ranging from students to program leadership to raise awareness about moral injury and provide tools to combat it.

**Methods:**

This 75-minute interactive workshop explored moral injury through a health care lens. It included components of lecture, case-based learning, small-group discussion, and individual reflection. Participants completed anonymous postworkshop evaluations, providing data on satisfaction and intention to change practice. We used descriptive statistics to analyze the quantitative data and applied content analysis to the qualitative data.

**Results:**

The workshop was presented at two local academic conferences. Data were collected from 34 out of 60 participants, for a response rate of 57%. Ninety-seven percent of participants felt the workshop helped them define and identify moral injury and was a valuable use of their time, as well as indicating they would apply the information learned in their daily life. One hundred percent would recommend the workshop to a friend or colleague. Almost half felt they could implement strategies to address moral injury after participating in the workshop.

**Discussion:**

This workshop proved to be a valuable tool to define and discuss moral injury. The materials can be adapted to a broad audience.

## Educational Objectives

By the end of this workshop, learners will be able to:

1. Define moral injury and relevant vocabulary.
2. Identify instances of moral injury and its effects on those around them.
3. Develop strategies to combat moral injury.

## Introduction

We experience stress when our morals are violated or when we feel pressured to choose between upholding one moral over another. Moral injury is defined differently by different scholars, but all agree it comprises feelings of guilt, despair, shame, and/or helplessness from having one's morals transgressed.^1–5^ The founding clinician scientists of moral injury include Jonathan Shay, William Nash, Brett Litz, and Shira Maguen.^5^ Moral injury was first described in military personnel presenting with trauma that, unlike post-traumatic stress disorder, was not a mental illness.^5,6^ These personnel described betraying their morals while following orders and committing and witnessing atrocities that they felt powerless to prevent.^2,3^ While moral injury has been well described in the literature for military personnel, it is not a well-studied or widely reported topic in the medical field.^1,4,7^ Koenig and colleagues developed the Moral Injury Symptom Scale, which measures 10 dimensions of moral injury including betrayal, guilt, and loss of meaning/purpose, and determined an objective threshold at which family, social, and occupational functioning was impacted. While many of these feelings may contribute to burnout, defined as exhaustion of physical or emotional strength or motivation usually as a result of prolonged stress or frustration, moral injury goes a step beyond burnout as it relates to a specific event. For moral injury to occur, two conditions must be present. First, there must be a troubling event that occurs. Second, this event must violate or transgress morals important to us. With these criteria in mind, instances of racism or macroaggressions may serve as triggers of moral injury if both criteria are met.

The medical field is characterized by stressful circumstances, and moral dilemmas often arise. Trainees and health care professionals must manage critical situations while under pressure to perform well. They witness or are subject to behaviors or actions that transgress their morals, perpetrated by members of the care team, patients, or patients’ families. Health care providers also report experiencing moral injury when the medical system (including insurance and documentation requirements) prevents them from providing high-quality equitable health care.^4,8^

The COVID-19 pandemic produced an unprecedented level of moral injury for health care providers.^9^ Some providers in the early stages of the pandemic put themselves and their families in danger caring for patients with suboptimal protection when personal protective equipment was scarce. When medical supplies were in demand, health care providers were sometimes forced to choose who they would offer what care to.^10^

We used the term *moral injury* to search *MedEdPORTAL.* The search yielded 10 articles. None of these 10 directly addressed the concept of moral injury, nor did they present examples with tools to combat it. When we performed a similar search on PubMed and limited results to the last 5 years, almost 1,500 articles were available. These articles were screened, and while many defined and explained the concept of moral injury, they did not utilize case-based examples to teach moral injury and empower participants the way we sought to.

In this workshop, we introduce the concept of moral injury, particularly as viewed through the lens of health care. We then create a space to discuss cases of moral injury in the medical field and encourage discussion to develop tools to combat moral injury. The workshop is designed for health care professionals, specifically, students, residents, fellows, faculty, and interprofessional health care members attending the annual Stanford School of Medicine Diversity and Inclusion Forum (DIF), to raise awareness about moral injury and provide tools to combat it.^6^

The workshop was developed over the course of 10 months as we completed the Stanford Leadership in Advancing Diversity (LEAD) Program. This program was developed by the Pediatrics Department at Stanford University and has been published.^11^ All participants in the LEAD Program are required to design and present a workshop on a topic of their choosing at the Stanford School of Medicine DIF. In selecting a topic, we chose moral injury because of its relative novelty and the lack of formal education on it. Despite the lack of education, the topic is extremely relatable as many of us can identify situations of moral injury that leave us with an uneasy feeling. This workshop first defines this novel concept, helps to identify it, and finally develops tools to combat or mitigate it.

## Methods

### Workshop

This 75-minute workshop was initially developed for students, residents, fellows, faculty, and interprofessional health care members attending the Stanford School of Medicine DIF,^11^ held annually at Stanford University and free to all university members. Presenters were asked to submit potential presentation topics ahead of the conference, and the final topics were then chosen by a selection committee. We developed this workshop as part of the Stanford LEAD Program and delivered it at the DIF. The workshop was also delivered separately, in the same format, to pediatric residents during their weekly educational luncheon session. With alterations in case-based examples, the workshop could be tailored to different audiences and given with more expansive or restrictive time allotments.

Workshop speakers and facilitators could be any team member facile with the concepts of moral injury and the application of the proposed tools within the workshop. There were no prior training requirements needed to facilitate the workshop. For our team, the facilitators included medical residents, medical fellows, attending physicians, and administrators. Six speakers or facilitators participated in the delivery of the workshop during the DIF. These six speakers were individuals completing the Stanford LEAD Program. When the workshop was presented, there were three to four breakout groups, and each group had one to two facilitators. The workshop could be delivered with fewer than six speakers. However, the number of speakers/facilitators is determined by the number of participants and the number of breakout groups, as each group requires at least one facilitator. When the workshop was initially presented at the DIF, roles for those involved in leading the workshop were assigned beforehand and ranged from leading didactics to hosting small-group sessions to leading case-based discussions (Appendix A). During implementation, small-group sizes ranged from four to 10 participants.

Due to the COVID-19 pandemic, the workshop was conducted in a virtual format using Zoom videoconferencing. One facilitator was designated to monitor the chat for comments and questions throughout the entirety of the 75-minute workshop. The workshop began with a didactic session focused on defining moral injury and terminology relevant to the workshop discussion as well as the goals and objectives for the session. Included in this portion of the workshop was a worksheet focused on identifying one's core values and which values were most important to each individual (Appendix B). After establishing a framework of concepts and terms within which to work and having identified values important to each participant, the facilitators took the audience through real-world examples. Through these examples, the concepts of moral injury were applied, and the transgressed values in each case were identified. Next, the audience was divided into small groups via breakout rooms, with each small group being assigned at least one facilitator. In the first small-group session, the previously provided examples were discussed. After this small-group discussion, the audience was brought back into the main room where the facilitators and audience volunteers summarized the key points surrounding the small-group discussion. Following the large-group discussion, there was a second breakout room session where the focus became identifying personal examples of moral injury and sharing them in a small-group setting. Then, the small groups reconvened as a larger audience, and volunteers shared the examples discussed with facilitators in a forum for open discussion.

The next component of the workshop focused on a didactic component where tools and strategies were identified to help combat moral injury. Following this didactic component, there was a third and final small-group session hosted via breakout rooms where the previously discussed examples of moral injury were dissected further to identify ways to prevent or combat the transgressions that had been identified. Again, after small-group discussions, the groups reconvened as a larger audience and shared the highlights of their collective discussions. This marked the conclusion of the workshop, Evaluations in the form of a postworkshop survey (Appendix C) were then collected virtually from all participants via an online survey. We did not perform preworkshop surveys.

The same sequence of events was used when the workshop was delivered during the noontime education session for Stanford pediatric residents, held in a conference room on the Stanford University campus. There were no variations in case-based examples required for this target audience. During this administration of the workshop, there were four facilitators. The number of facilitators for this administration was determined by those members from the original group who happened to be available on this day, proving that the workshop can be administered with fewer than the six facilitators used initially.

The workshop timeline can be found in Appendix A. A PowerPoint presentation was used to guide the workshop (Appendix D) as well as a handout for all facilitators to help lead case-based discussions (Appendix E).

### Workshop Evaluation

At the conclusion of the workshop, participants were asked to complete an anonymous survey regarding satisfaction with the workshop and intention to change practice. The evaluation featured six items scored on a 5-point Likert scale (1 = *strongly disagree,* 5 = *strongly agree*) and four open-ended questions and finished by asking participants if they would recommend the workshop to a friend or colleague. In addition, demographic information was collected regarding race/ethnicity and role in the health care system.

We developed the survey in accordance with the learning objectives set forth at the beginning of the workshop. Given the novel nature of moral injury, the survey was not based on any prior publications. There was no pilot testing, and no validity evidence was collected.

### Analysis

We used descriptive statistics to analyze the quantitative data (percentage of participants who strongly agreed or agreed). A single investigator (Connor Arquette) analyzed the open-ended questions using content analysis, which was then verified by two independent reviewers (Valerie Peicher and Rebecca Blankenburg). Frequency counts were determined by the number of times a category was mentioned by the participants. These frequency counts were used to identify the top three categories/themes, which are reported in the Results, below.

Percentages were calculated for each Likert-scale question and for the final question asking if participants would recommend the workshop to a friend.

### Institutional Review Board

The workshop was reviewed by the Institutional Review Board of Stanford University (Protocol Number 63203) and was granted exemption as it was deemed not to qualify as human subjects research.

## Results

The workshop was presented twice—once at the 2021 Stanford School of Medicine DIF in a virtual format and once in a noon conference for pediatric residents at Stanford. Twenty-four of 50 participants at the DIF and 10 of 10 participants at the pediatric noon conference responded. Responders comprised residents, fellows, attending physicians, and program administrators from residency programs within Stanford and other US and international institutions.

Ninety-four percent of participants felt the workshop met its intended learning objectives, and 97% felt it was a valuable use of their time. Furthermore, 94% of participants found the supplemental materials useful, and 97% felt they would apply the information learned in their daily life. One hundred percent of participants responded that they would recommend the workshop to a friend or colleague (Table 1).

**Table 1.**
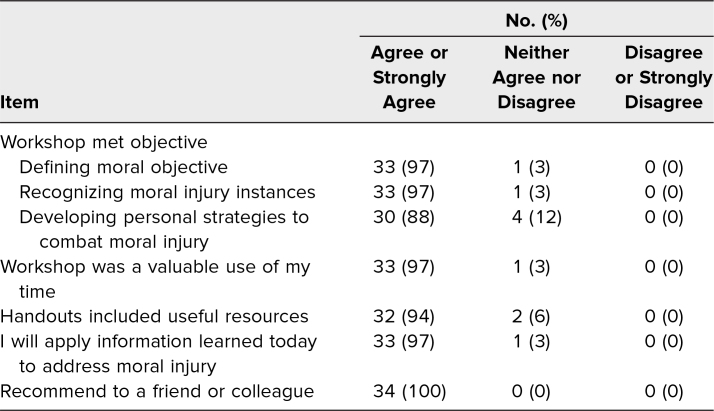
Participant Ratings of Workshop Experience (*N* = 34)

Participants were asked to share two things they learned from the workshop, highlight potential barriers that might prevent implementation of strategies to mitigate moral injury, and identify what aspect they enjoyed most about the workshop. Participants’ areas of learning from the workshop included (1) definition/recognition of moral injury, (2) identification of personal values, and (3) implementation of strategies to minimize moral injury. The most common barriers identified included time, resources, hierarchical structure, and fear of retaliation. The most enjoyed portions of the workshop were the case-based discussions and examples (Table 2).

**Table 2.**
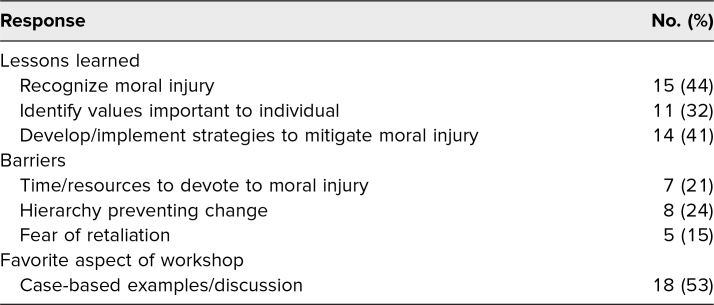


## Discussion

We felt this topic was timely, especially at the height of the pandemic. Education regarding this novel area and empowering health care providers to identify moral injury and begin to fight back against it were the main motivators for developing the workshop. Additionally, many of us had been directly affected by the Stanford vaccine case and experienced moral injury as a result of it.

Throughout the workshop's development, the topic's novelty served as a challenge. We had to differentiate moral injury from topics like burnout. Whereas burnout is defined as exhaustion of physical or emotional strength or motivation as a result of prolonged stress, moral injury goes a step beyond because it relates to a specific, identifiable event. We developed case-based examples to highlight this difference.

We delivered the workshop in two separate settings, once via Zoom during the pandemic and once in person. There was a wide range of audience members with varying types of provider backgrounds. While this was a strength in terms of the values and diversity of experience that each person brought to the small-group discussions, it also served as a challenge when attempting to direct the small groups to understanding the clinical nature of the cases provided. Additionally, the virtual format utilized for the initial presentation of this workshop was challenging as it mitigated some of the emotional discussion inherent to this topic.

Participants rated the workshop highly, with all of them stating they would recommend the workshop to a friend. All but one participant either agreed or strongly agreed the workshop was a valuable use of their time. The one participant who did not agree or strongly agree responded in a neutral fashion.

This workshop provides actionable steps that health care providers can take when they encounter moral injury, a common occurrence in health care and more common for individuals who are underrepresented in health care. We found that our audiences were highly engaged and reflective during the session. The small-group discussions and case examples were reported to be most impactful for our participants. Furthermore, the top themes identified in the open-ended questions answered by participants aligned with the educational objectives set forth in the workshop. This further illustrates that the participants felt the workshop met its intended objectives.

In administering the workshop, we learned it could be delivered effectively with far fewer facilitators than initially utilized. This is important as it allows future groups to deliver the workshop more easily. Additionally, it became evident during administration of the workshop that most participants were unfamiliar with the term *moral injury* prior to taking part. Thus, more time was needed to accurately define the topic and ensure all participants understood the vocabulary before proceeding.

While the evaluations demonstrated that the workshop was well received, its design was not without limitations. The workshop was administered in only two settings, and we did not stratify our data based on demographics.

Our evaluation focused on participants’ self-perceived outcomes after completing the workshop. There was no direct assessment of the participants’ ability to meet the intended objectives of the workshop. Thus, while participants felt the workshop achieved its intended objectives, there was no objective measurement to correlate with this perception. Ideally, the workshop could feature pre- and postworkshop data collection to demonstrate how it influences participants’ ability to meet the stated objectives. The workshop did not feature a preworkshop evaluation of participants, thus limiting the finality of the conclusions that could be drawn.

Finally, the data we gathered was about planned behavior change, not actual behavior change. To gather our data, we administered volunteer surveys, so we also acknowledge any volunteer bias that could potentially confound the results we obtained. Future studies should monitor for long-term changes implemented as a result of participating in the workshop.

Following administration of the workshop, the following lessons were learned:

• Moral injury impacts all of us, for different reasons, based upon what we value.
• Identifying our values helps us better identify when they have been violated.
• Identifying instances of moral injury and the violated values allows us to develop strategies to combat moral injury.
• There may be personal strategies, systems-level strategies, or both that help mitigate instances of moral injury.

Participants in this workshop can share it with members of their respective groups or institutions. In doing so, they should seek to differentiate between moral injury and burnout and to identify circumstances of moral injury. By recognizing events that cause moral injury, participants can label them appropriately and be poised to initiate discussion surrounding the values violated. In identifying the effects of an event on those around them, participants can begin to develop personal and systems-level strategies to mitigate or prevent it from becoming a repeat occurrence. To make best use of the information in this workshop, participants must first define their own hierarchy of values. Using this set of values, participants can then interpret instances of moral injury through their own lens. In this publication, we have included a list of takeaways (Appendix F) to be distributed to participants following future implementations of the workshop.

The data presented highlight the development of a successful workshop to define moral injury, identify and discuss real-world examples, and provide participants with strategies to combat moral injury. As moral injury is increasingly recognized as a contributor to burnout for health care providers, the ability to identify and address these instances of moral trespass can not only empower health care providers with control over their own wellness but also help them to better connect with their patients, colleagues, and friends as they identify which values are most important to the involved parties. Future studies should explore ways to build time and psychological safety into training programs and work environments so individuals can reflect on the situations that cause moral injury and take actionable steps toward improvement.

## Appendices


Workshop Timeline.docxWorkshop Handout.docxWorkshop Evaluation.docxWorkshop PowerPoint.pptxFacilitator Guide.docxParticipant Takeaways.docx
*All appendices are peer reviewed as integral parts of the Original Publication.*
