# Supplementary material for: Moral Injury: How It Affects Us and Tools to Combat It
Source: MedEdPORTAL. 2023 Nov 3;19:11357. doi: 10.15766/mep_2374-8265.11357 (PMC10622333; doi:10.15766/mep_2374-8265.11357)
Supplement: Supplementary file 1 — Workshop Timeline.docxWorkshop Handout.docxWorkshop Evaluation.docxWorkshop PowerPoint.pptxFacilitator Guide.docxParticipant Takeaways.docx [file mep_2374-8265.11357-s001.zip › A. Workshop Timeline.docx]

Appendix A – Workshop Timeline

| **Time** | **Notes** | **Slide(s)** | **Speaker(s)** |
| --- | --- | --- | --- |
| Minutes 0-3 | Quick welcome, introduction to meeting as safe space  **Objectives**  **Remind participants that there is a practice guide** | 1-4 | Speaker 1 |
| Minutes 4-5 | Brief Didactic: Differentiating Moral Injury, Two Elements of Moral Injury | 5-6 | Speaker 2 |
| Minutes 6-11 | Brief Didactic: Define values | 7-9 | Speaker 3 |
| Minutes 12-15 |  | 10-12 | Speakers 4 & 5 |
| Minutes 15-24 | Facilitated Small Group #1 | 13-14 | ---------- |
| Minutes 24-25 | Return to main room | ---------- | ----------- |
| Minutes 25-33 | Large Group: Reflections | 15 | Speakers 4 & 5 |
| Minutes 33-35 |  | 16 | Speaker 3 |
| Minutes 35-38 | Facilitated Small Group #2 | 17 | ---------- |
| Minutes 38-39 | Return to main room | ----------- | ----------- |
| Minutes 39-48 | Brief Didactic: Stanford Vaccine Case | 18-24 | Speaker 6 |
| Minutes 48-52 | Actionable Steps - personal, system | 25-26 | Speakers 2 & 6 |
| Minutes 52-61 | Facilitated Small Group #3 | 27 | Speaker 1 |
| Minutes 61-62 | Return to main room | ---------- | ----------- |
| Minutes 63-65 | Final reflections, references, contact info | 28-32 | Speaker 5 |
