# Supplementary material for: Moral Injury: How It Affects Us and Tools to Combat It
Source: MedEdPORTAL. 2023 Nov 3;19:11357. doi: 10.15766/mep_2374-8265.11357 (PMC10622333; doi:10.15766/mep_2374-8265.11357)
Supplement: Supplementary file 1 — Workshop Timeline.docxWorkshop Handout.docxWorkshop Evaluation.docxWorkshop PowerPoint.pptxFacilitator Guide.docxParticipant Takeaways.docx [file mep_2374-8265.11357-s001.zip › B. Workshop Handout.docx]

**MORAL INJURY WORKSHOP – Participant Worksheet**

Moral injury occurs when we experience a troubling event that violates our values.

**CLARIFYING YOUR VALUES**

(Adapted From Tobias Lundgren’s Bull’s Eye Worksheet)

Values are not the same as goals. Values involve ongoing action; they are like directions we keep moving in, whereas goals are what we want to achieve along the way. Goals can be achieved or ‘crossed off’, whereas values are ongoing.

For example, if you want to be a loving, caring, supportive partner, that is a value: it involves ongoing action. In contrast, if you want to get married, that’s a goal - it can be ‘crossed off’ or achieved.

If you want a better job, that’s a goal. Once you’ve got it - goal achieved. But if you want to fully apply yourself at work, contribute your best, and engage fully in what you’re doing, that’s a value: it involves ongoing action.

**YOUR VALUES:** *What sort of person do you want to be? What personal strengths or qualities do you want to develop?*

Choose 10 values that are most important to you. You can choose from the sample list below or you are welcome to come up with your own values.

- Autonomy
- Family
- Honesty
- Courage
- Adventure
- Intelligence
- Professionalism
- Patience
- Diversity
- Justice
- Equity
- Advocacy
- Work-life balance
- Empathy
- Leadership
- Meaningful work
- Generosity
- Integrity
- Teamwork
- Meaningful relationships
- Prosperity
- Endurance
- Respect
- Humor
- Faith
- Pride in your work

**THE BULL’S EYE:** Draw out a target or bull’s eye. Take the 10 values that you have chosen and mark an X on a Bull’s Eye where you think you are in terms of living by your values. Put an X closer to the Bull’s eye if you feel like you are living fully by a chosen value and put an X farther away if you inconsistently living by that value. For each X placed, label this with which specific value this corresponds to.

**RECOGNIZING MORAL INJURY**

Read the case examples below. In your small groups, discuss what values may have been transgressed.

Case 1: You are a nurse in a pediatric patient’s room with his Spanish speaking mother. He has had head trauma and had steri-strips and bandages on his forehead. A resident comes in without introduction and proceeds to remove the dressings from the patient’s head. The patient cries out in shock/pain. The mother looks at you confused and concerned that her son is in pain. The other resident walks out; you apologize to the mother.

Case 2: You are receiving sign out as the overnight fellow in the cardiothoracic intensive care unit. Your patient is a 70 yo white man who indicates that he is a retired police officer. During sign out, you introduce yourself as the fellow on call, to which the patient responds “I thought all of you Black girls were out rioting”. None of the other members of the care team speak up.

**REFLECTING BACK ON YOUR VALUES**

1. Looking back on your bullseye worksheet, identify a few key values that are important to you
2. Has there been a time when you acted in a way or felt compelled to change your actions in a way that was contradictory to your values?
3. Spend a few minutes thinking of an example of a troubling event and share this in your group. As a group, identify values that were transgressed

**Reference**

1. Lundgren T, Luoma JB, Dahl J, Strosahl K, Melin L. The Bull's-Eye Values Survey: A Psychometric Evaluation. Cognitive Behavior Pract. 2012:19;518-526.
