# Supplementary material for: Moral Injury: How It Affects Us and Tools to Combat It
Source: MedEdPORTAL. 2023 Nov 3;19:11357. doi: 10.15766/mep_2374-8265.11357 (PMC10622333; doi:10.15766/mep_2374-8265.11357)
Supplement: Supplementary file 1 — Workshop Timeline.docxWorkshop Handout.docxWorkshop Evaluation.docxWorkshop PowerPoint.pptxFacilitator Guide.docxParticipant Takeaways.docx [file mep_2374-8265.11357-s001.zip › C. Workshop Evaluation.docx]

Moral Injury: How it affects us and tools to combat it

Evaluation Form

*Please complete this brief evaluation to give us your feedback so we can continue to improve this workshop for future audiences*

Workshop met the following objectives:

1. Define moral injury
   1. Strongly disagree
   2. Disagree
   3. Neither disagree or agree
   4. Agree
   5. Strongly agree
2. Recognize instances of moral injury and its impact on us and others
   1. Strongly disagree
   2. Disagree
   3. Neither disagree or agree
   4. Agree
   5. Strongly agree
3. Develop personal strategies to combat moral injury
   1. Strongly disagree
   2. Disagree
   3. Neither disagree or agree
   4. Agree
   5. Strongly agree
4. Workshop was a valuable use of my time:
   1. Strongly disagree
   2. Disagree
   3. Neither disagree or agree
   4. Agree
   5. Strongly agree
5. Handouts include useful resources:
   1. Strongly disagree
   2. Disagree
   3. Neither disagree or agree
   4. Agree
   5. Strongly agree
6. I will apply information learned today to address moral injury:
   1. Strongly disagree
   2. Disagree
   3. Neither disagree or agree
   4. Agree
   5. Strongly agree
7. What two things will you implement as a result of this workshop? (Free response)
8. What do you see as potential barriers to applying what you have learned? (Free response)
9. What did you like best about the workshop? (Free response)
10. Any other comments/suggestions for improvement? (Free response)
11. Would you recommend this workshop to a friend/colleague?
    1. Yes
    2. No
12. Which of the following best describes your racial or ethnic identification? (Select all that apply)
    1. American Indian or Alaska Native
    2. Asian or Asian American
    3. Black or African American
    4. Hispanic, Latino, or of Spanish origin
    5. Native Hawaiian or Other Pacific Islander
    6. Non-Hispanic White
    7. Prefer to self-describe
    8. Prefer not to answer
13. Which of the following describes your sexual orientation?
    1. Bisexual
    2. Gay
    3. Heterosexual
    4. Lesbian
    5. Pansexual
    6. Prefer to self-describe (free response)
    7. Prefer not to answer
