# Supplementary material for: Moral Injury: How It Affects Us and Tools to Combat It
Source: MedEdPORTAL. 2023 Nov 3;19:11357. doi: 10.15766/mep_2374-8265.11357 (PMC10622333; doi:10.15766/mep_2374-8265.11357)
Supplement: Supplementary file 1 — Workshop Timeline.docxWorkshop Handout.docxWorkshop Evaluation.docxWorkshop PowerPoint.pptxFacilitator Guide.docxParticipant Takeaways.docx [file mep_2374-8265.11357-s001.zip › D. Workshop PowerPoint.pptx]

## Slide 1
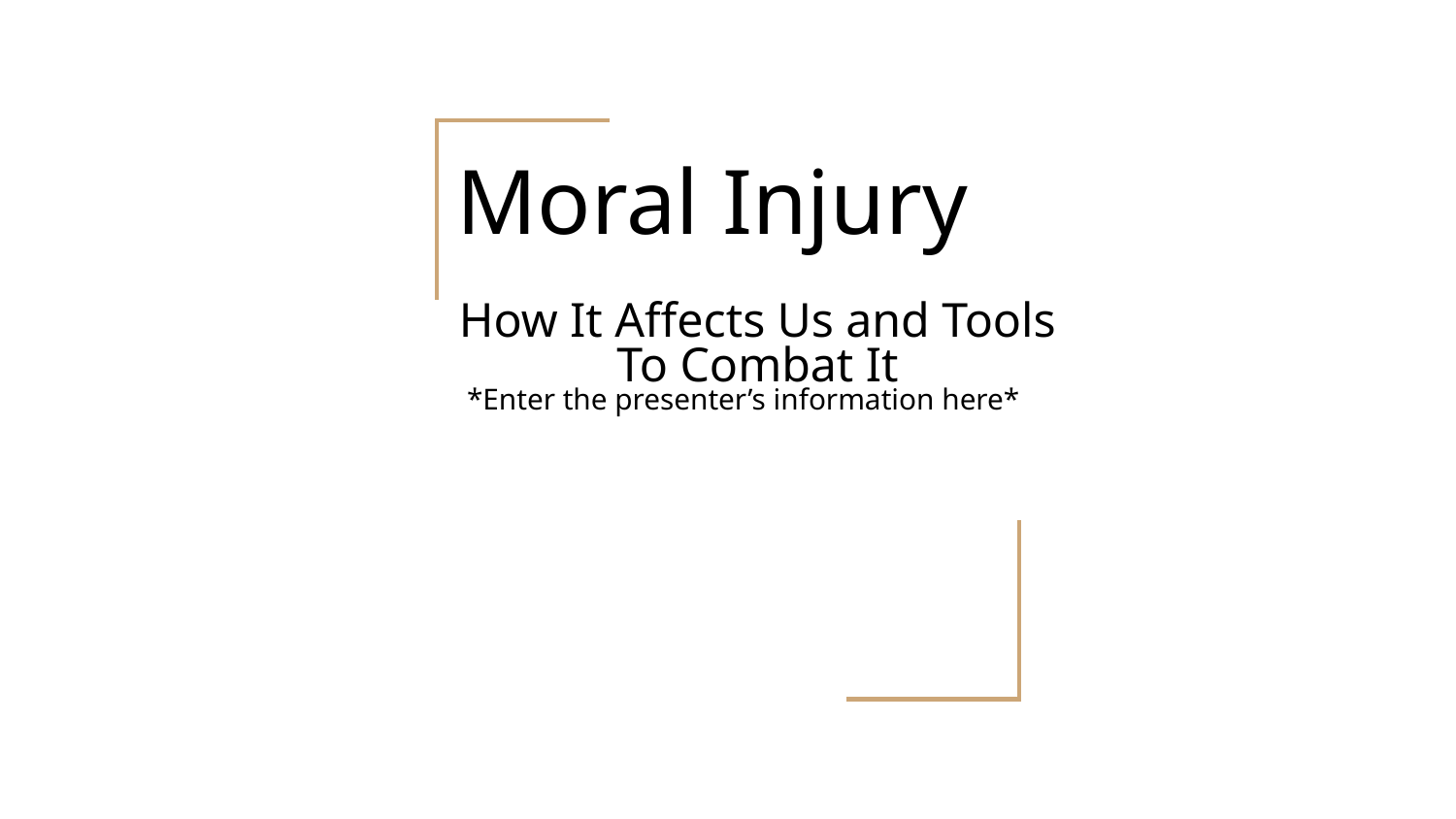

# Moral Injury
How It Affects Us and Tools To Combat It
*Enter the presenter’s information here*

## Slide 2
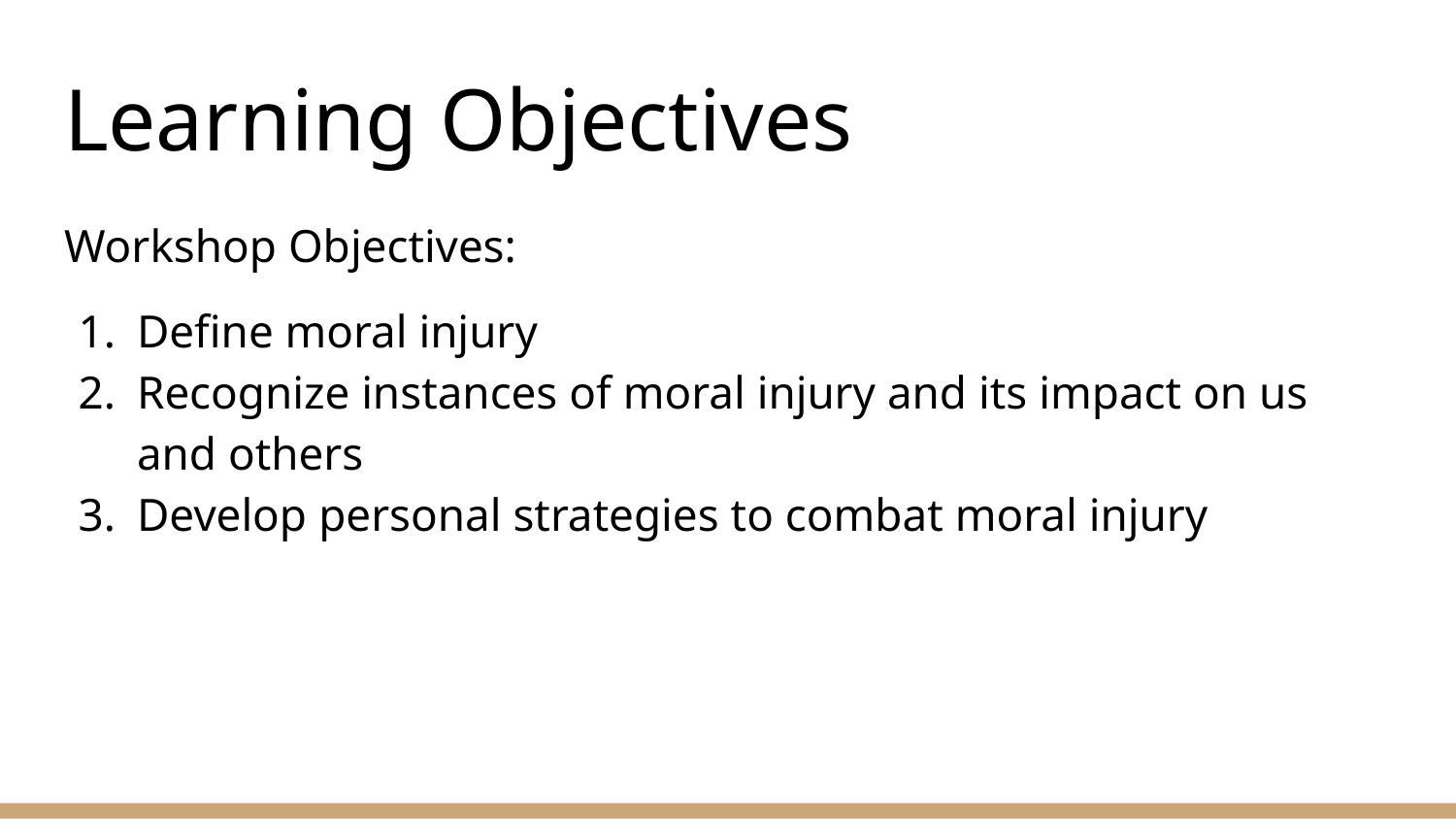

# Learning Objectives
Workshop Objectives:
Define moral injury
Recognize instances of moral injury and its impact on us and others
Develop personal strategies to combat moral injury

## Slide 3
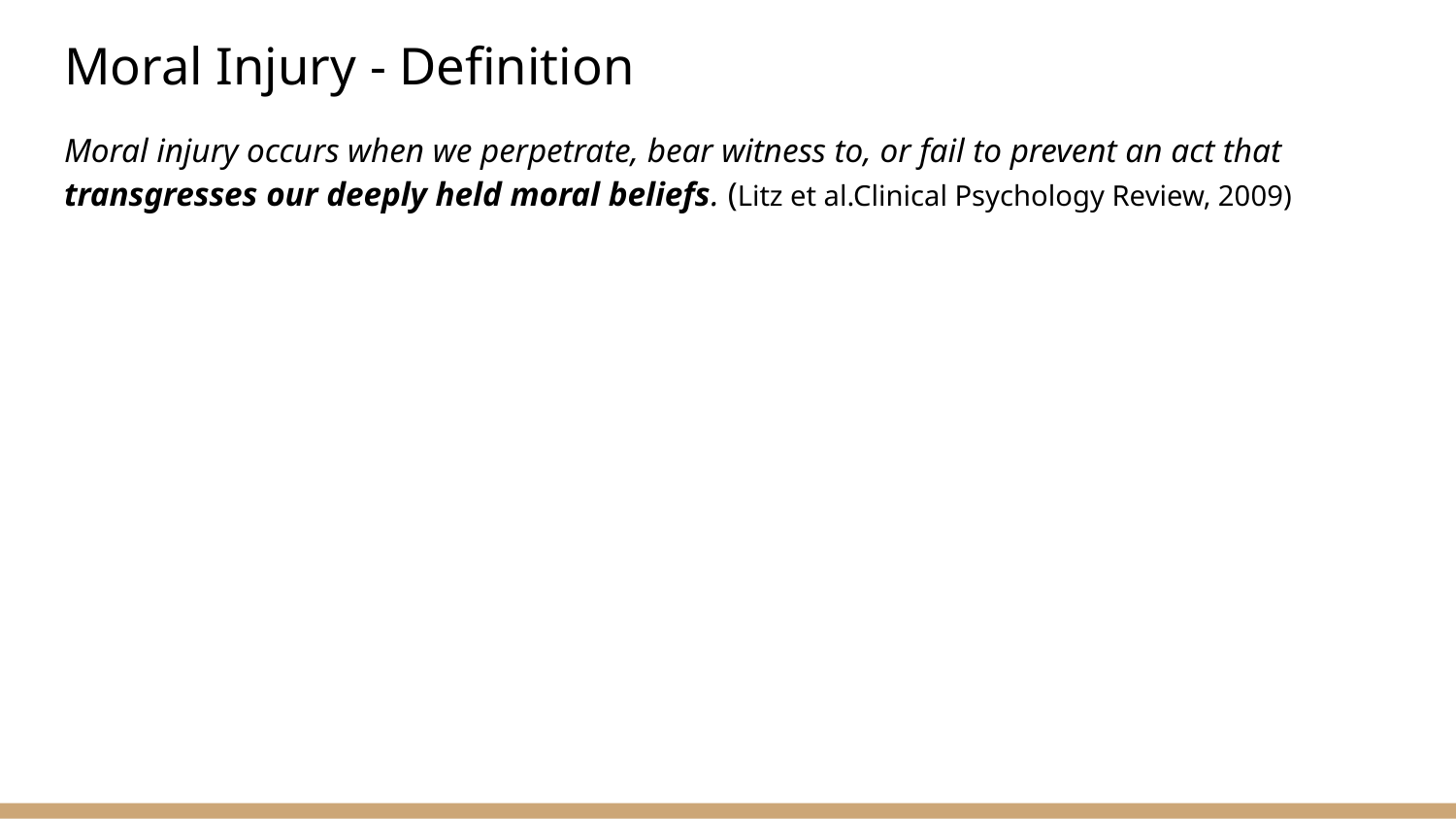

# Moral Injury - Definition
Moral injury occurs when we perpetrate, bear witness to, or fail to prevent an act that transgresses our deeply held moral beliefs. (Litz et al.Clinical Psychology Review, 2009)

## Slide 4
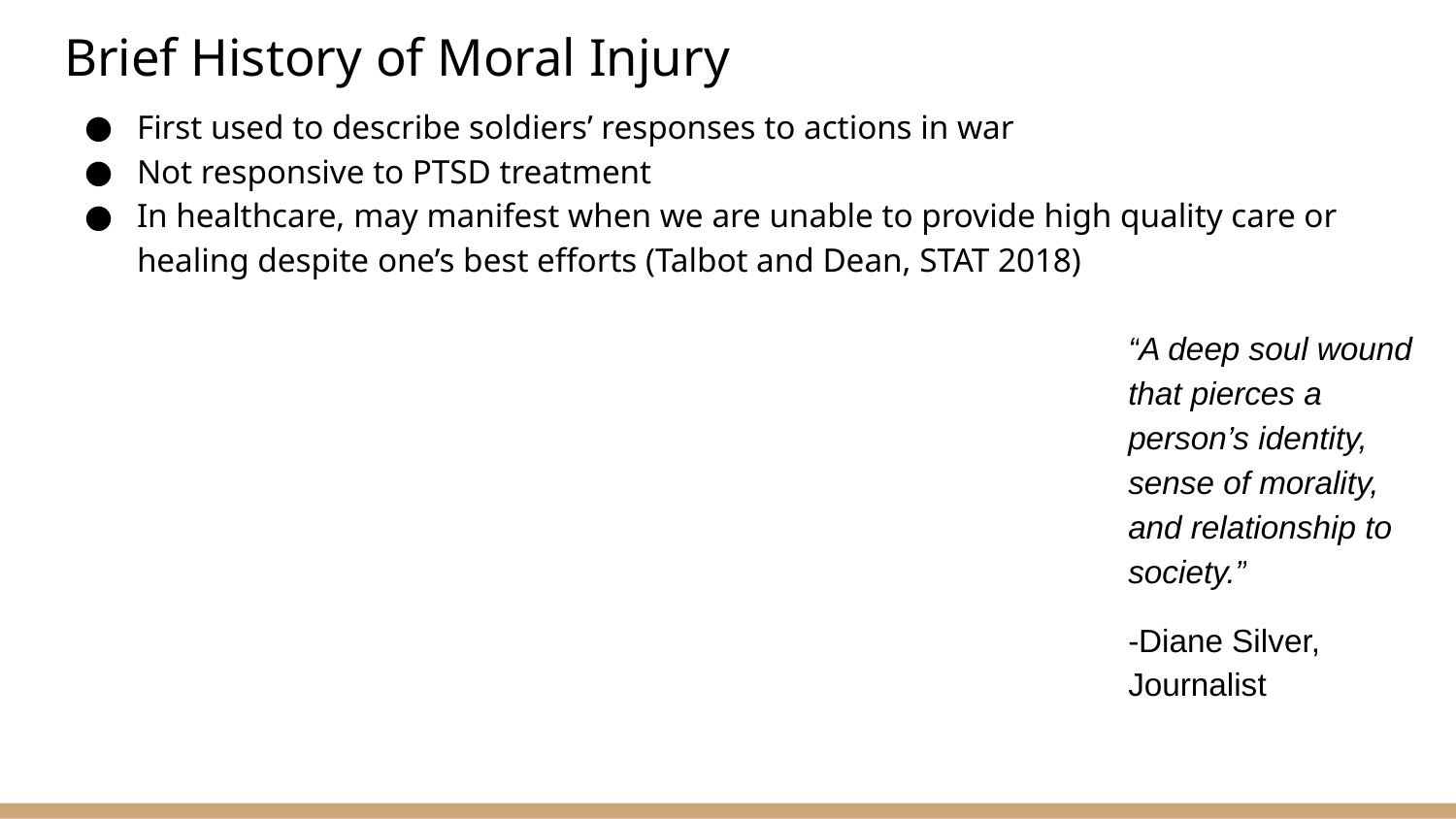

# Brief History of Moral Injury
First used to describe soldiers’ responses to actions in war
Not responsive to PTSD treatment
In healthcare, may manifest when we are unable to provide high quality care or healing despite one’s best efforts (Talbot and Dean, STAT 2018)
“A deep soul wound that pierces a person’s identity, sense of morality, and relationship to society.”
-Diane Silver, Journalist

## Slide 5
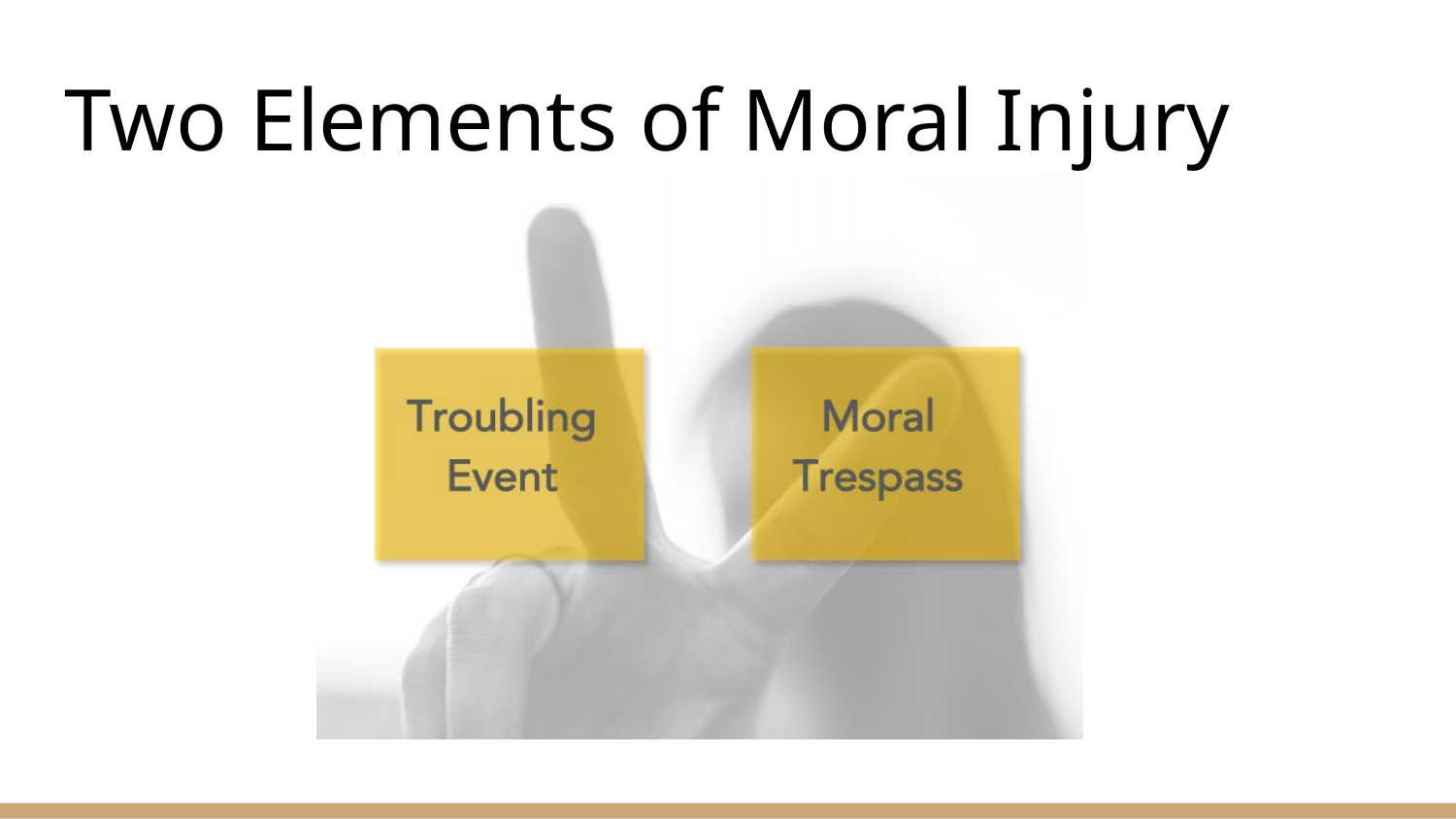

# Two Elements of Moral Injury

## Slide 6
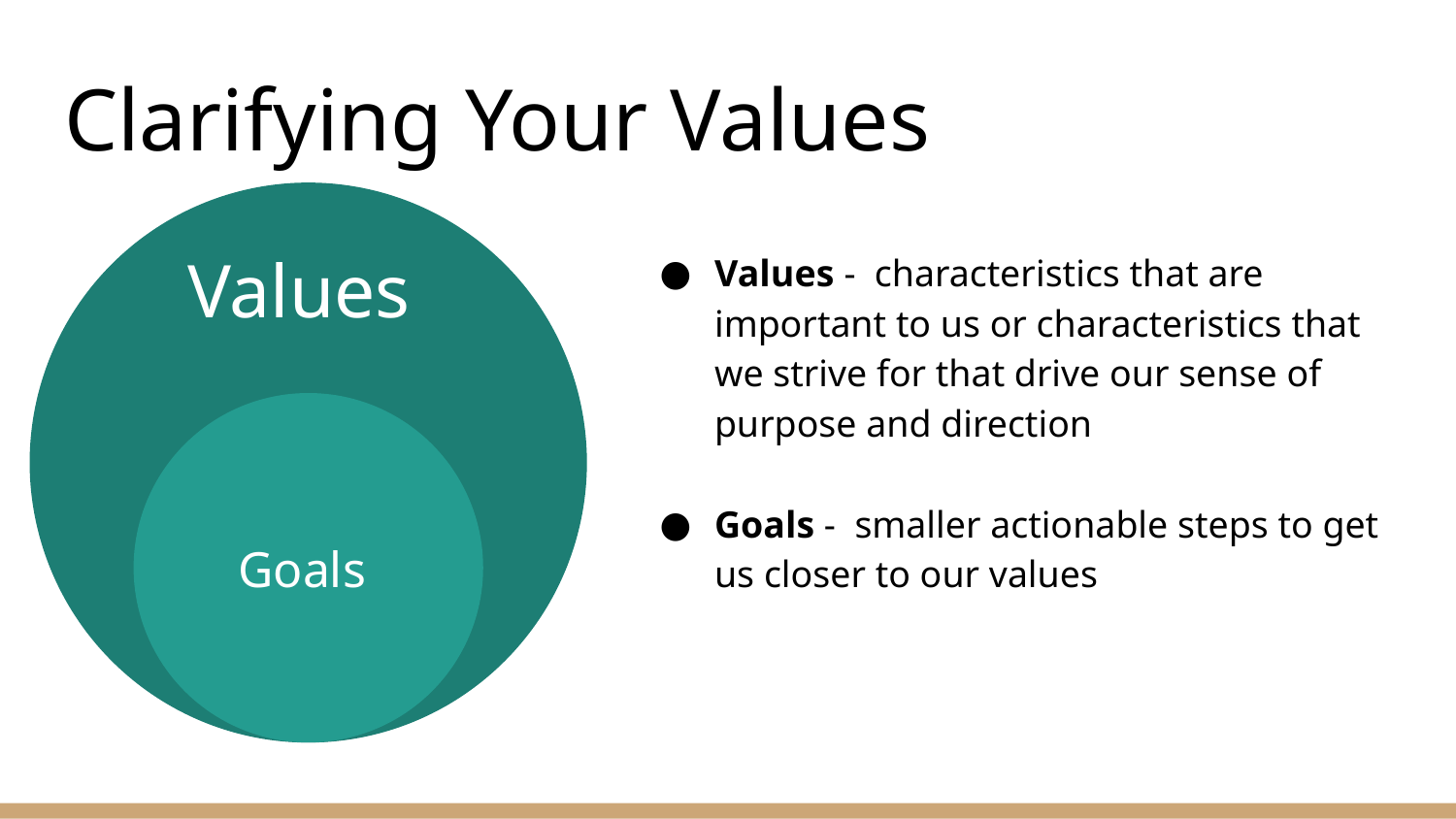

# Clarifying Your Values
Values - characteristics that are important to us or characteristics that we strive for that drive our sense of purpose and direction
Goals - smaller actionable steps to get us closer to our values
Values
Goals

## Slide 7
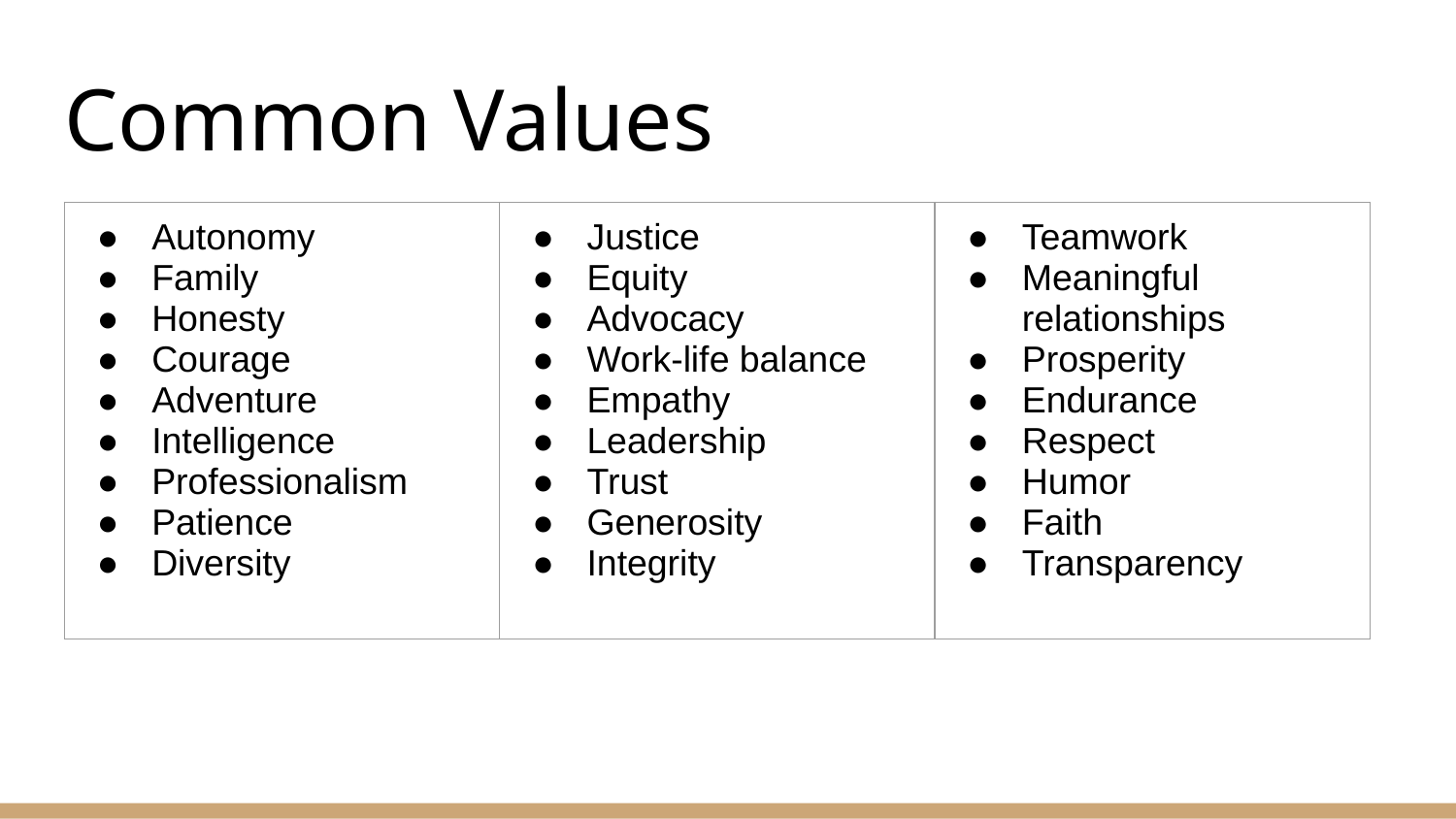

# Common Values
| Autonomy Family Honesty Courage Adventure Intelligence Professionalism Patience Diversity | Justice Equity Advocacy Work-life balance Empathy Leadership Trust Generosity Integrity | Teamwork Meaningful relationships Prosperity Endurance Respect Humor Faith Transparency |
| --- | --- | --- |

## Slide 8
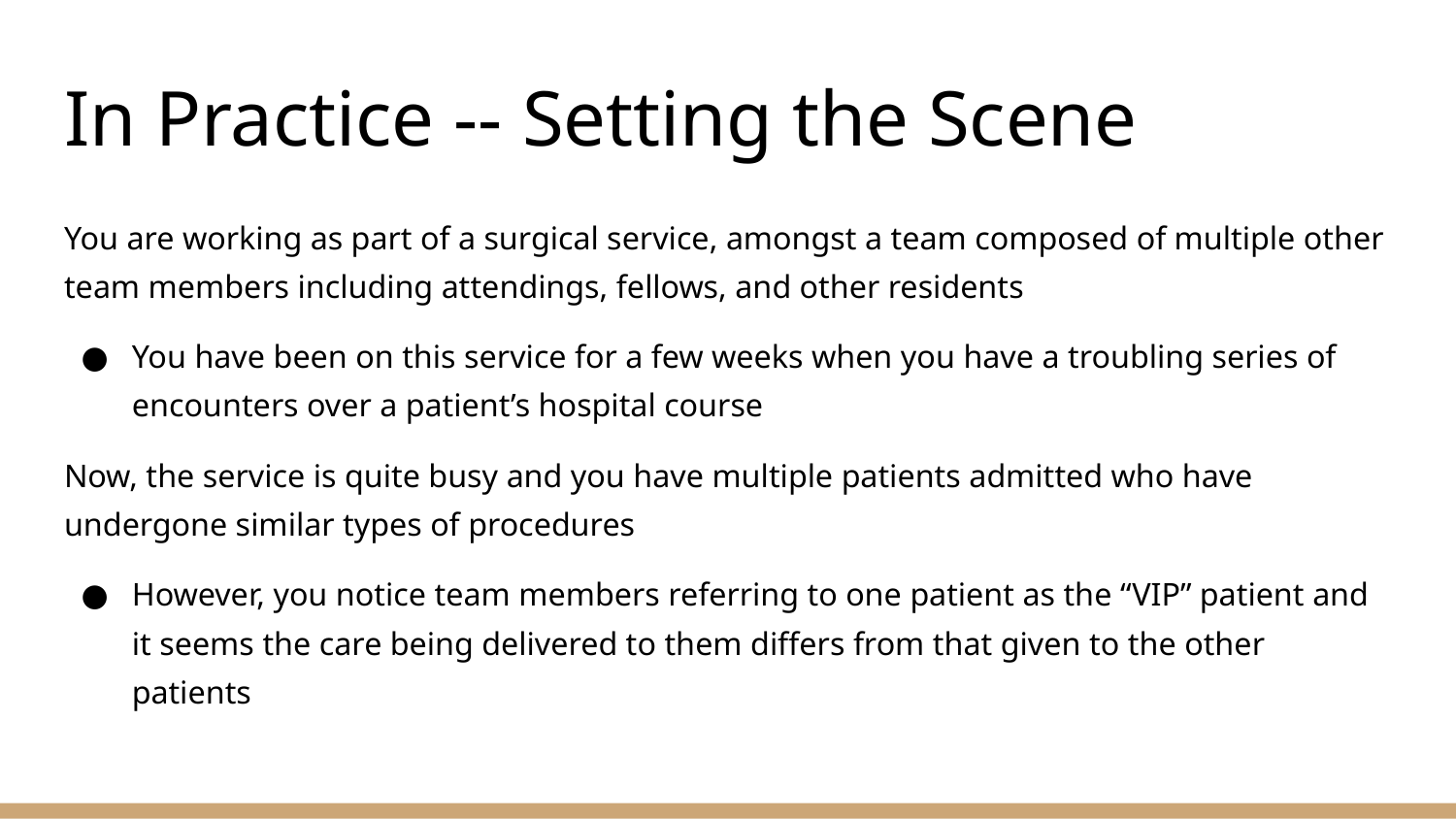

# In Practice -- Setting the Scene
You are working as part of a surgical service, amongst a team composed of multiple other team members including attendings, fellows, and other residents
You have been on this service for a few weeks when you have a troubling series of encounters over a patient’s hospital course
Now, the service is quite busy and you have multiple patients admitted who have undergone similar types of procedures
However, you notice team members referring to one patient as the “VIP” patient and it seems the care being delivered to them differs from that given to the other patients

## Slide 9
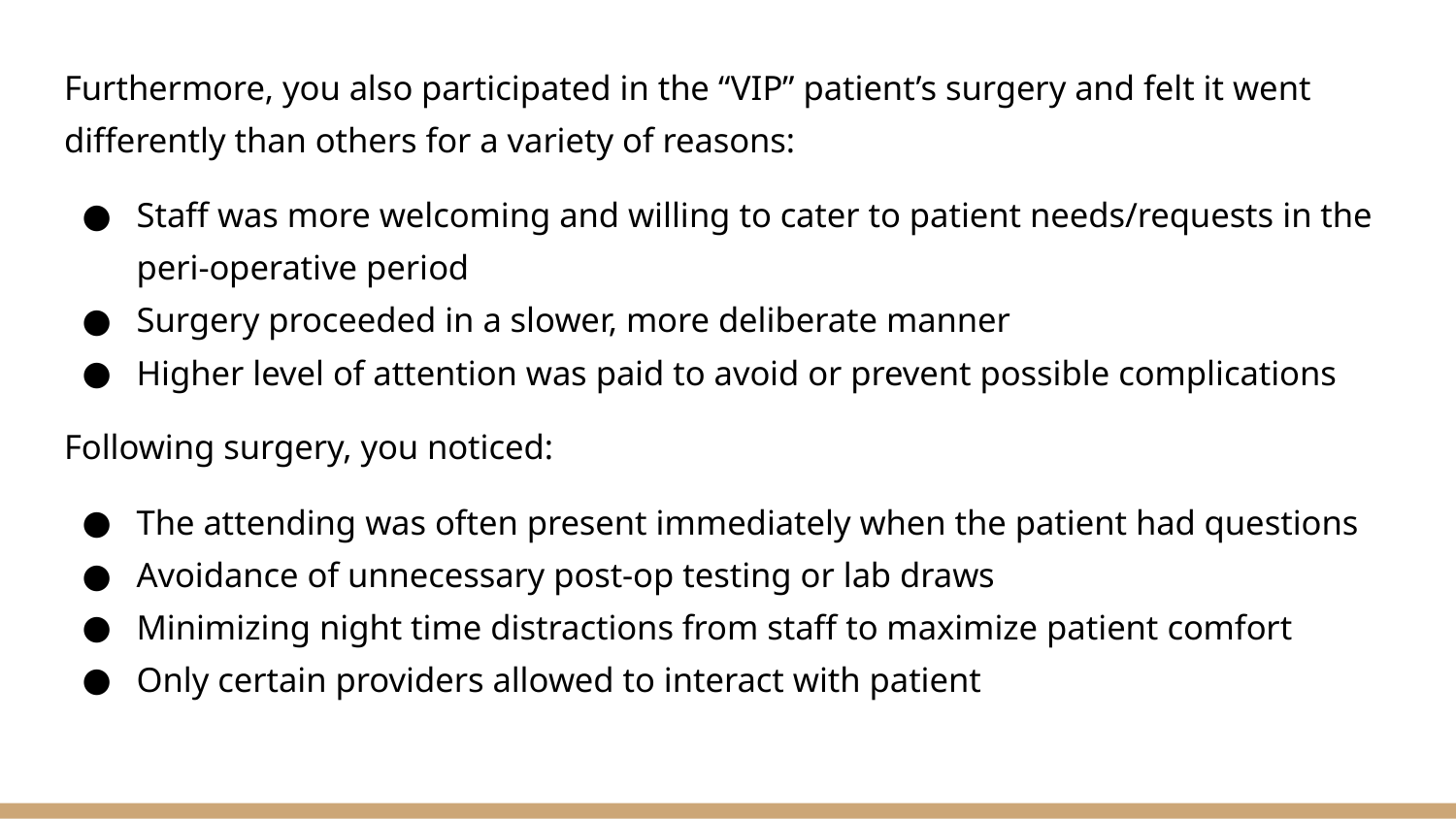

Furthermore, you also participated in the “VIP” patient’s surgery and felt it went differently than others for a variety of reasons:
Staff was more welcoming and willing to cater to patient needs/requests in the peri-operative period
Surgery proceeded in a slower, more deliberate manner
Higher level of attention was paid to avoid or prevent possible complications
Following surgery, you noticed:
The attending was often present immediately when the patient had questions
Avoidance of unnecessary post-op testing or lab draws
Minimizing night time distractions from staff to maximize patient comfort
Only certain providers allowed to interact with patient

## Slide 10
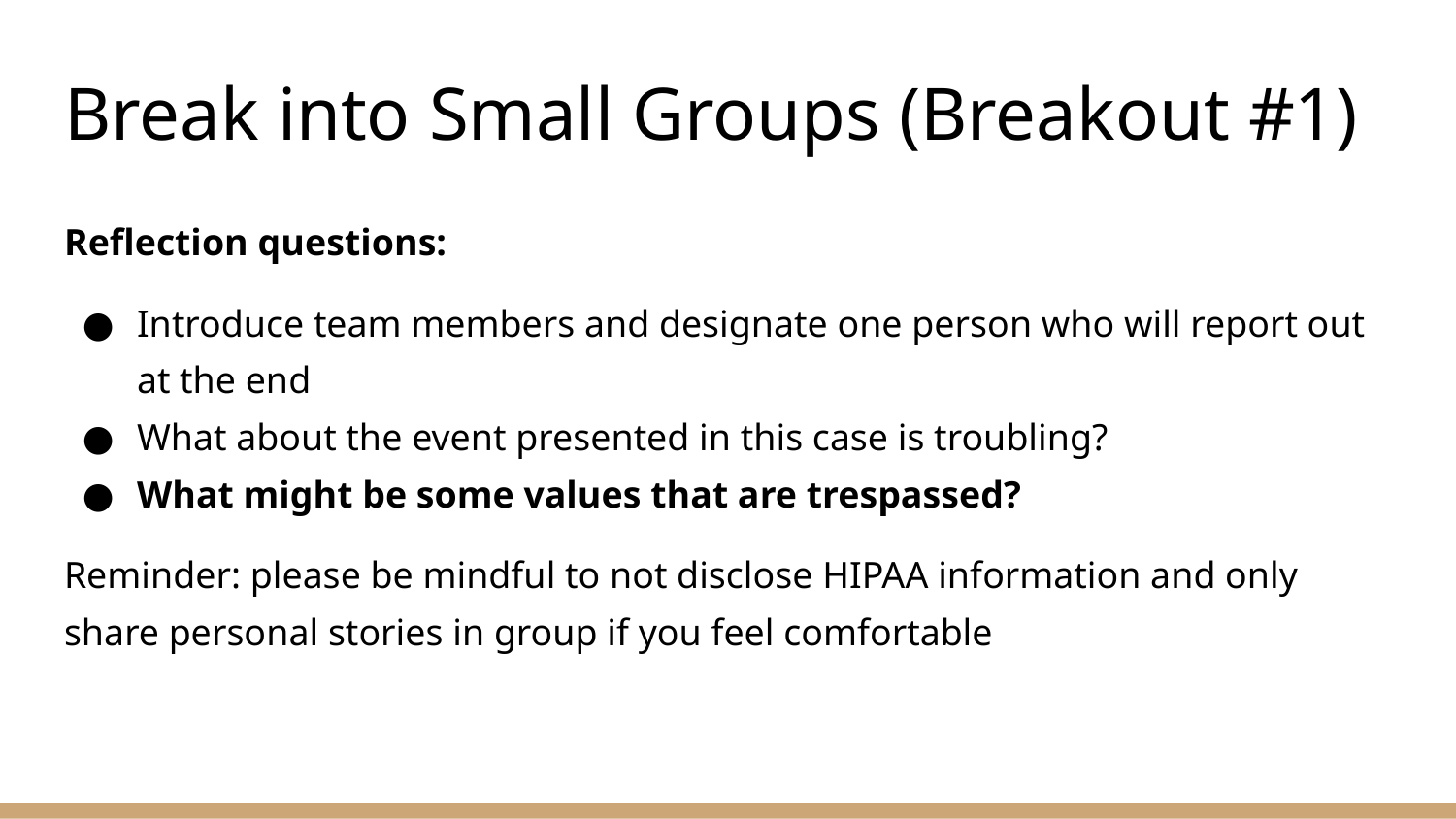

# Break into Small Groups (Breakout #1)
Reflection questions:
Introduce team members and designate one person who will report out at the end
What about the event presented in this case is troubling?
What might be some values that are trespassed?
Reminder: please be mindful to not disclose HIPAA information and only share personal stories in group if you feel comfortable

## Slide 11
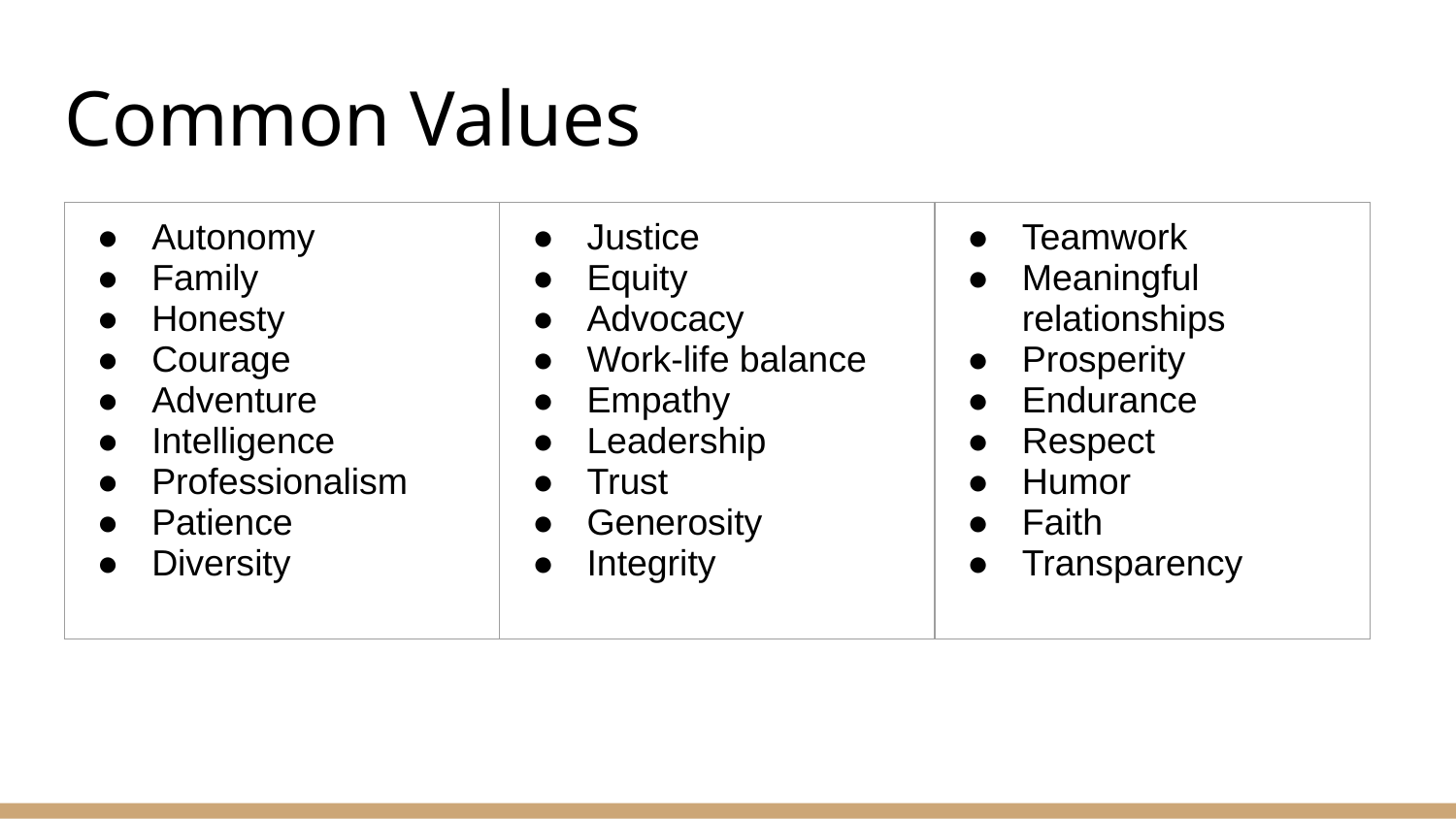

# Common Values
| Autonomy Family Honesty Courage Adventure Intelligence Professionalism Patience Diversity | Justice Equity Advocacy Work-life balance Empathy Leadership Trust Generosity Integrity | Teamwork Meaningful relationships Prosperity Endurance Respect Humor Faith Transparency |
| --- | --- | --- |

## Slide 12
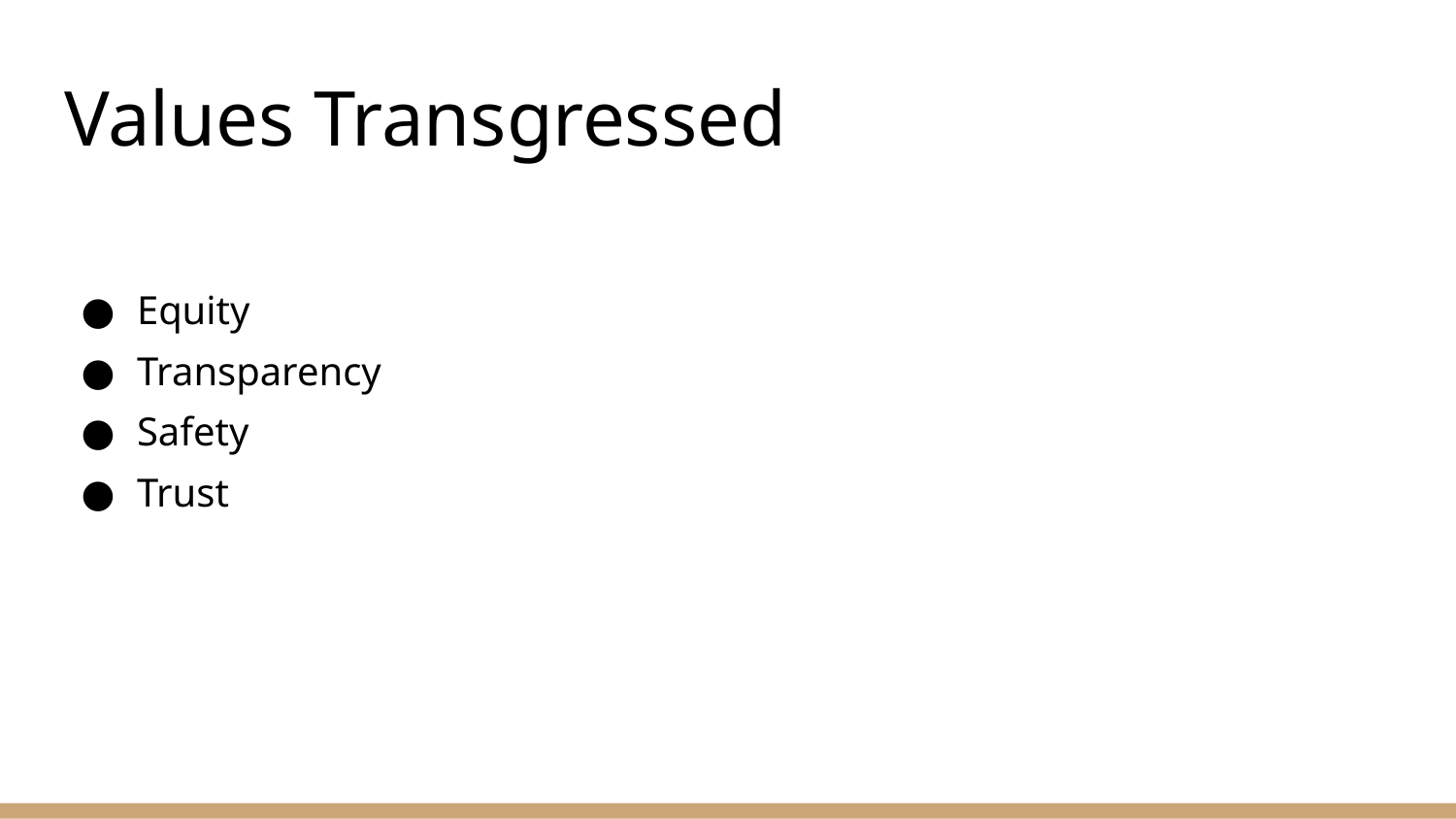

# Values Transgressed
Equity
Transparency
Safety
Trust

## Slide 13
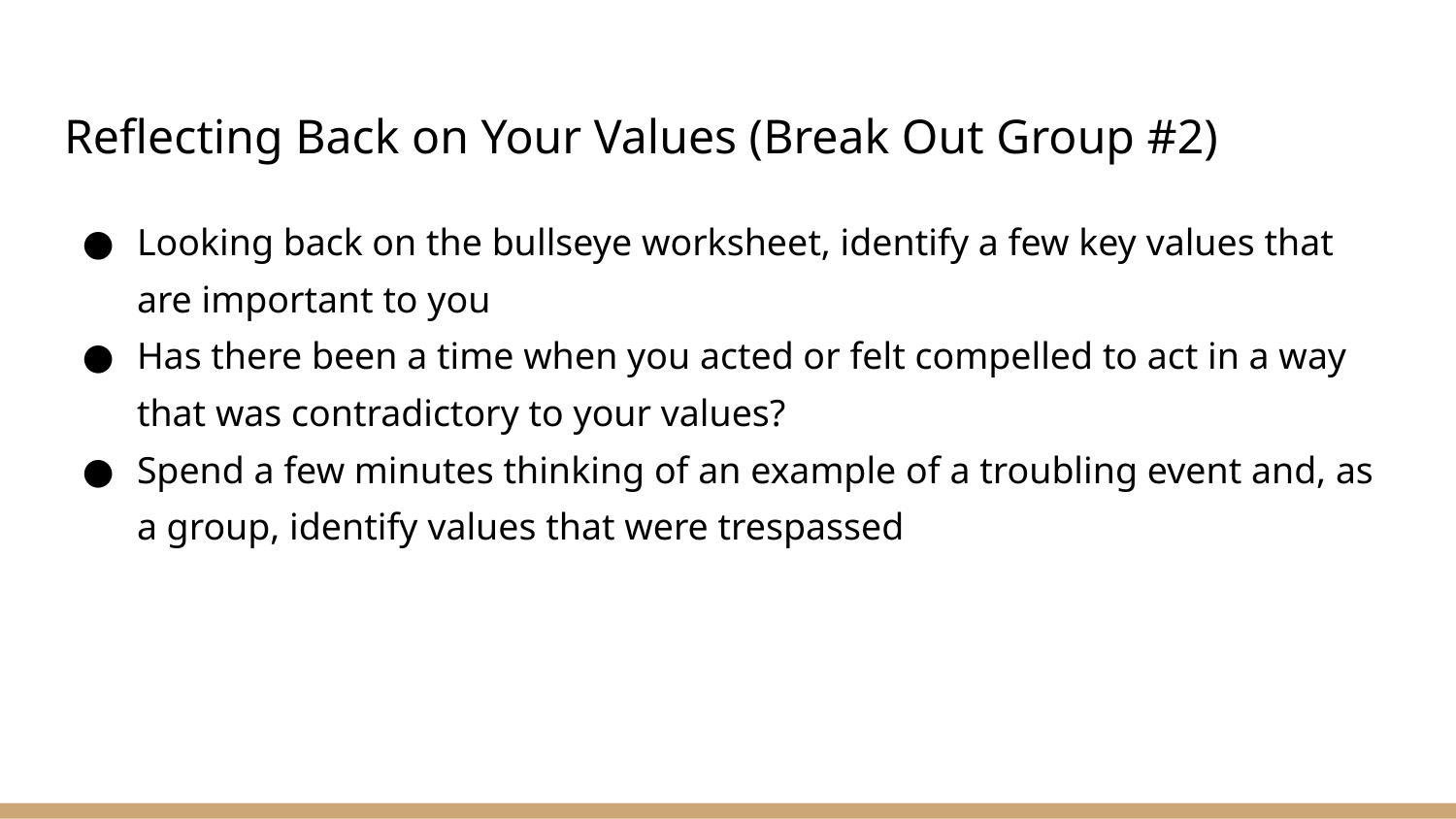

# Reflecting Back on Your Values (Break Out Group #2)
Looking back on the bullseye worksheet, identify a few key values that are important to you
Has there been a time when you acted or felt compelled to act in a way that was contradictory to your values?
Spend a few minutes thinking of an example of a troubling event and, as a group, identify values that were trespassed

## Slide 14
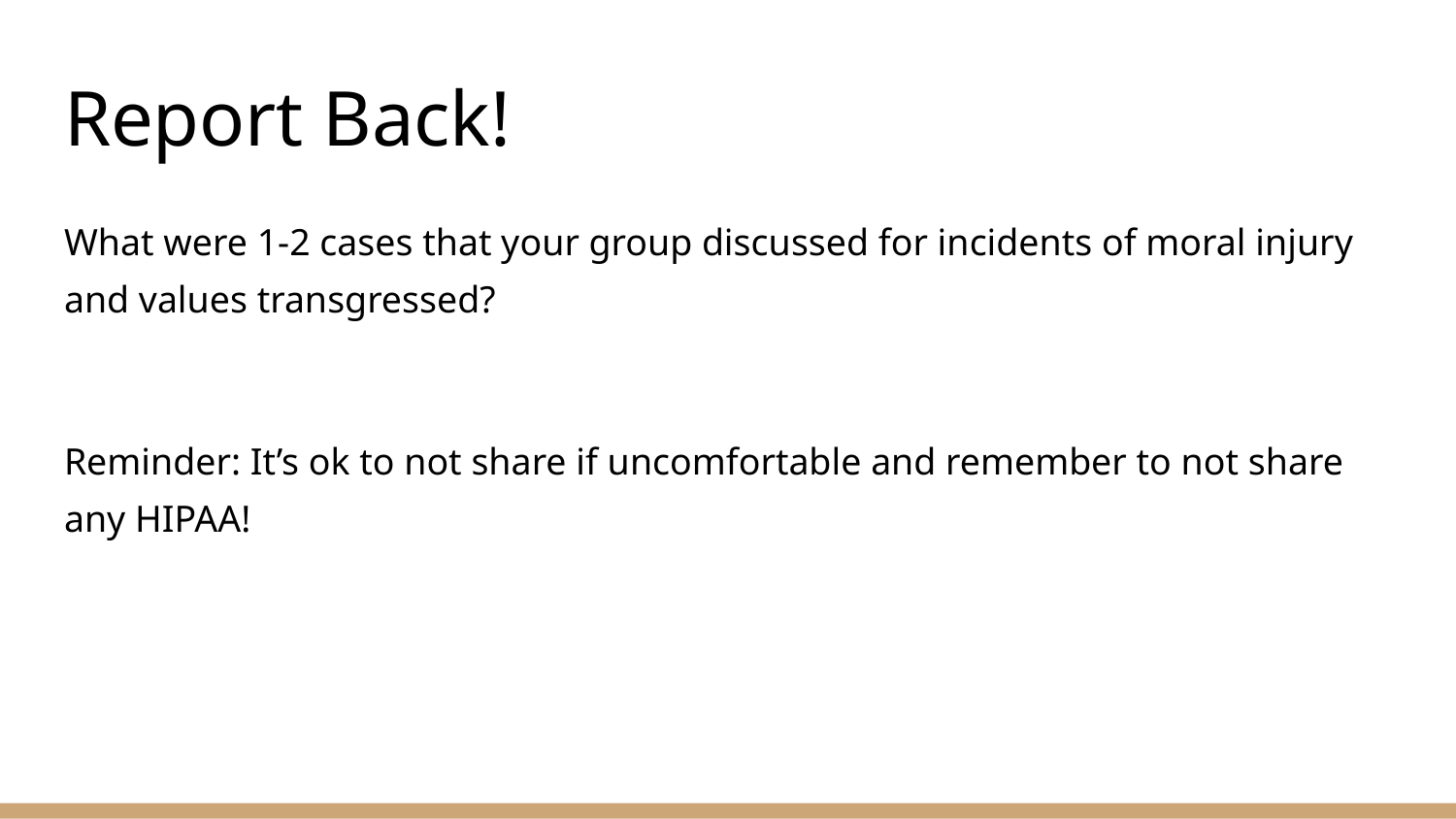

# Report Back!
What were 1-2 cases that your group discussed for incidents of moral injury and values transgressed?
Reminder: It’s ok to not share if uncomfortable and remember to not share any HIPAA!

## Slide 15
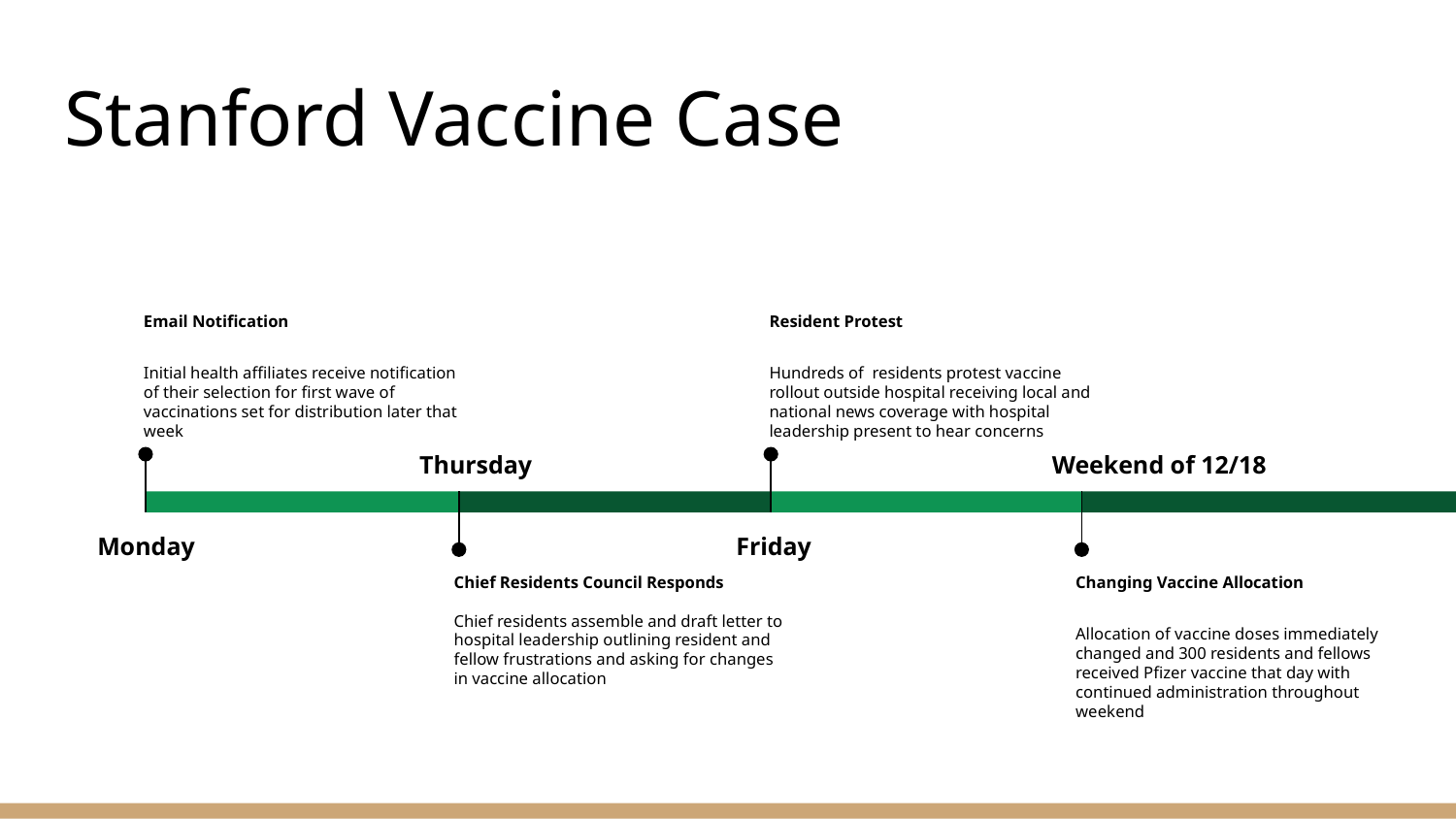

# Stanford Vaccine Case
Email Notification
Initial health affiliates receive notification of their selection for first wave of vaccinations set for distribution later that week
Monday
Resident Protest
Hundreds of residents protest vaccine rollout outside hospital receiving local and national news coverage with hospital leadership present to hear concerns
Friday
Thursday
Chief Residents Council Responds
Chief residents assemble and draft letter to hospital leadership outlining resident and fellow frustrations and asking for changes in vaccine allocation
Weekend of 12/18
Changing Vaccine Allocation
Allocation of vaccine doses immediately changed and 300 residents and fellows received Pfizer vaccine that day with continued administration throughout weekend

## Slide 16
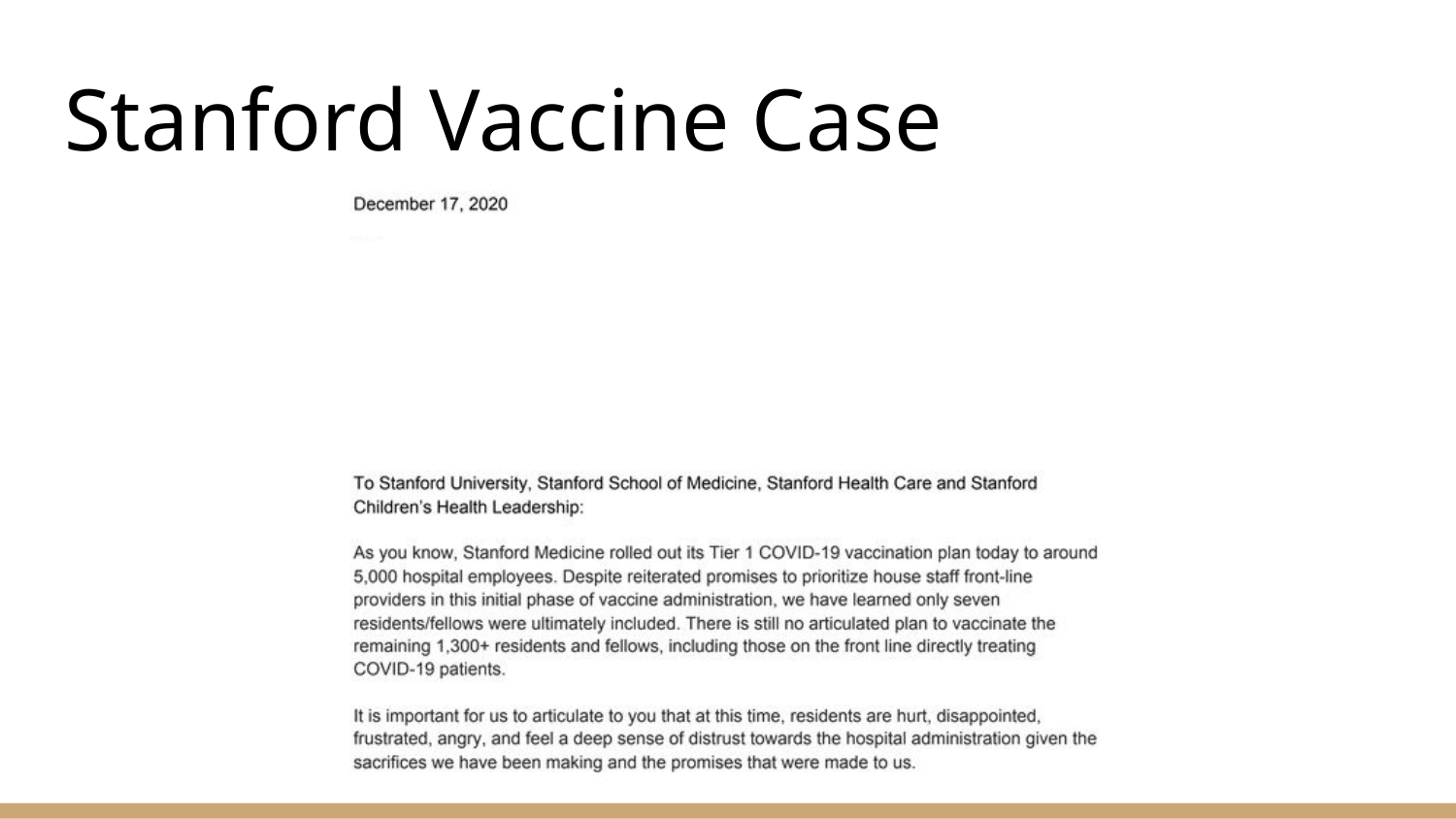

# Stanford Vaccine Case

## Slide 17
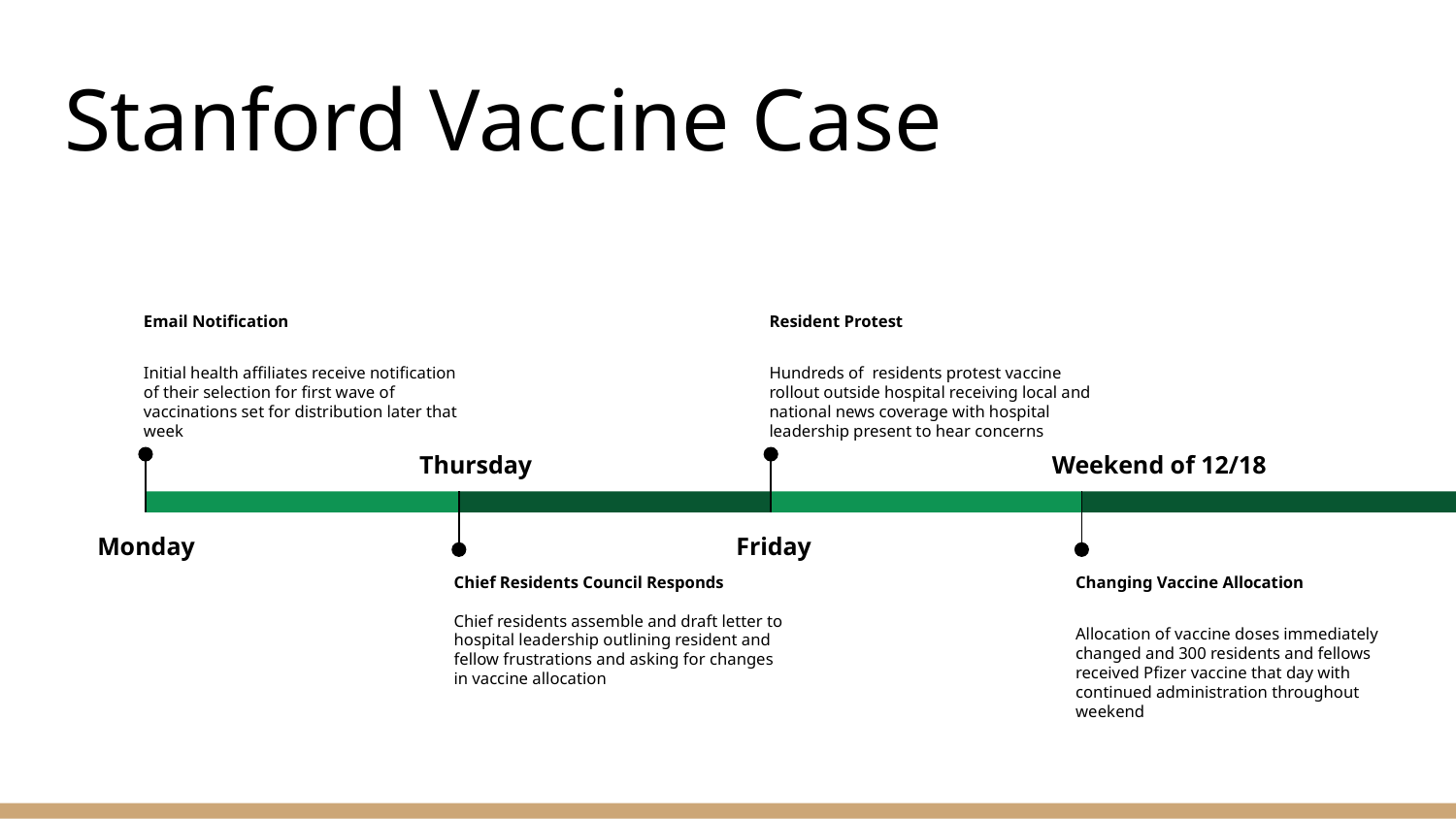

# Stanford Vaccine Case
Email Notification
Initial health affiliates receive notification of their selection for first wave of vaccinations set for distribution later that week
Monday
Resident Protest
Hundreds of residents protest vaccine rollout outside hospital receiving local and national news coverage with hospital leadership present to hear concerns
Friday
Thursday
Chief Residents Council Responds
Chief residents assemble and draft letter to hospital leadership outlining resident and fellow frustrations and asking for changes in vaccine allocation
Weekend of 12/18
Changing Vaccine Allocation
Allocation of vaccine doses immediately changed and 300 residents and fellows received Pfizer vaccine that day with continued administration throughout weekend

## Slide 18
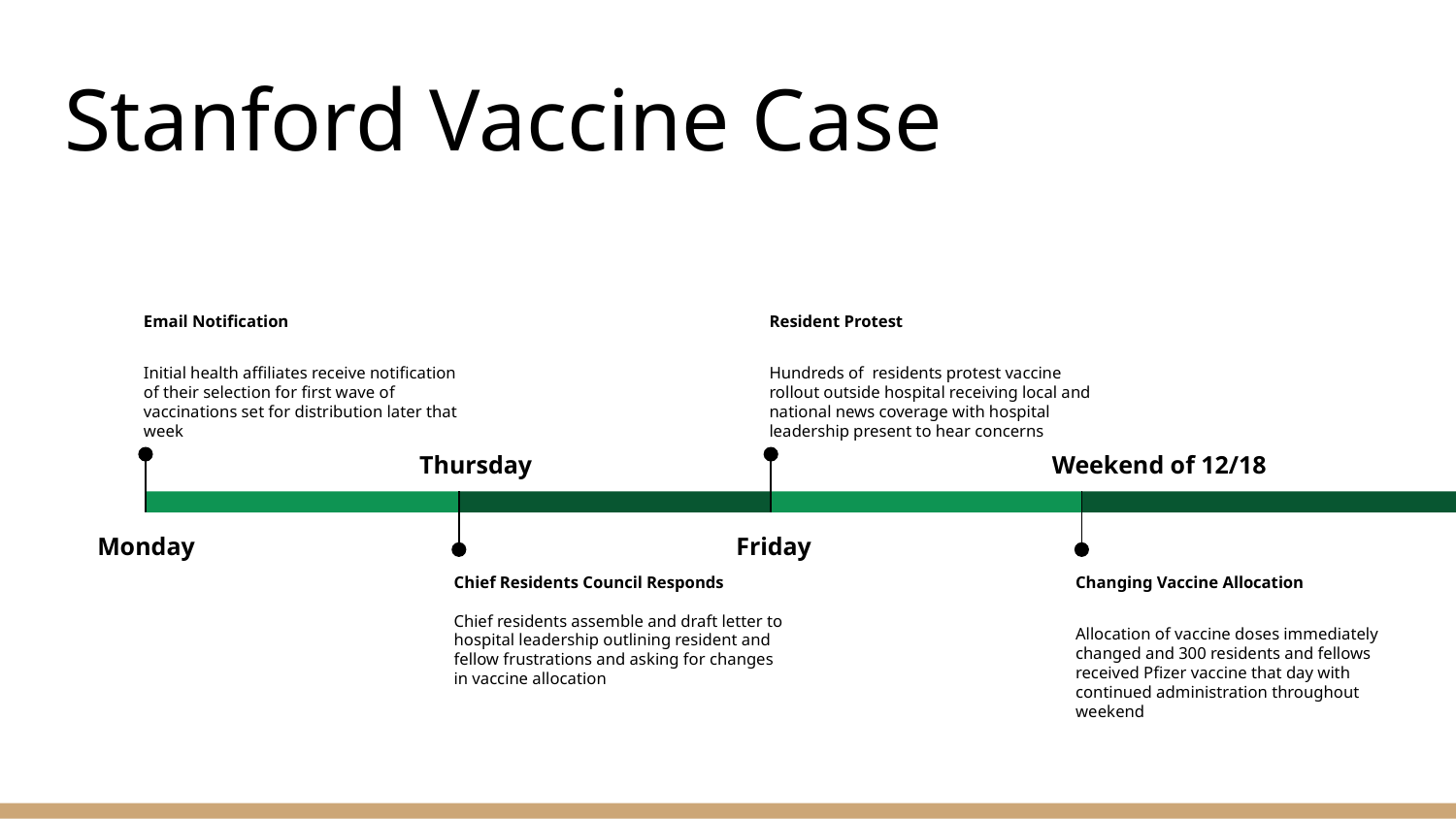

# Stanford Vaccine Case
Email Notification
Initial health affiliates receive notification of their selection for first wave of vaccinations set for distribution later that week
Monday
Resident Protest
Hundreds of residents protest vaccine rollout outside hospital receiving local and national news coverage with hospital leadership present to hear concerns
Friday
Thursday
Chief Residents Council Responds
Chief residents assemble and draft letter to hospital leadership outlining resident and fellow frustrations and asking for changes in vaccine allocation
Weekend of 12/18
Changing Vaccine Allocation
Allocation of vaccine doses immediately changed and 300 residents and fellows received Pfizer vaccine that day with continued administration throughout weekend

## Slide 19
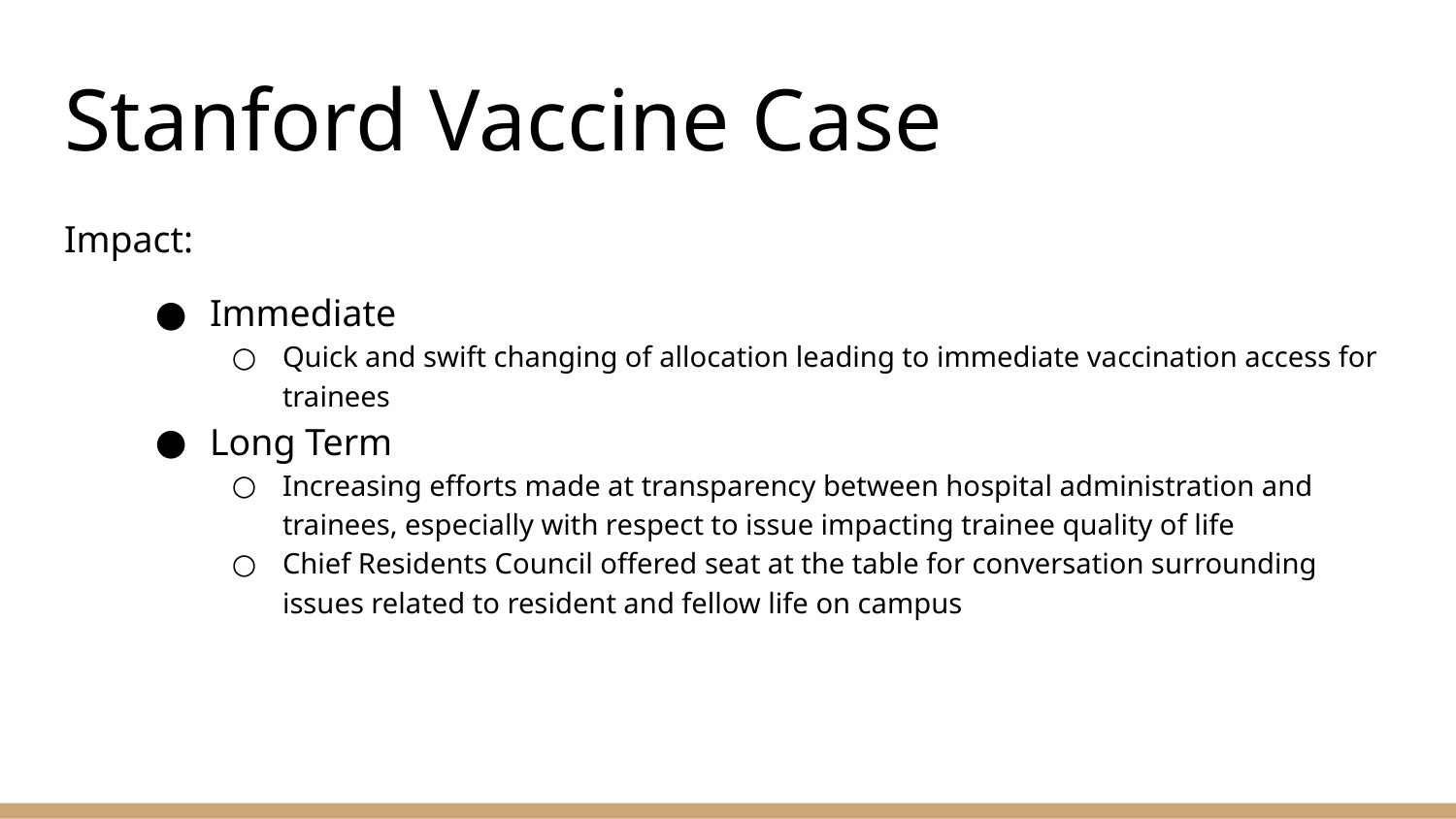

# Stanford Vaccine Case
Impact:
Immediate
Quick and swift changing of allocation leading to immediate vaccination access for trainees
Long Term
Increasing efforts made at transparency between hospital administration and trainees, especially with respect to issue impacting trainee quality of life
Chief Residents Council offered seat at the table for conversation surrounding issues related to resident and fellow life on campus

## Slide 20
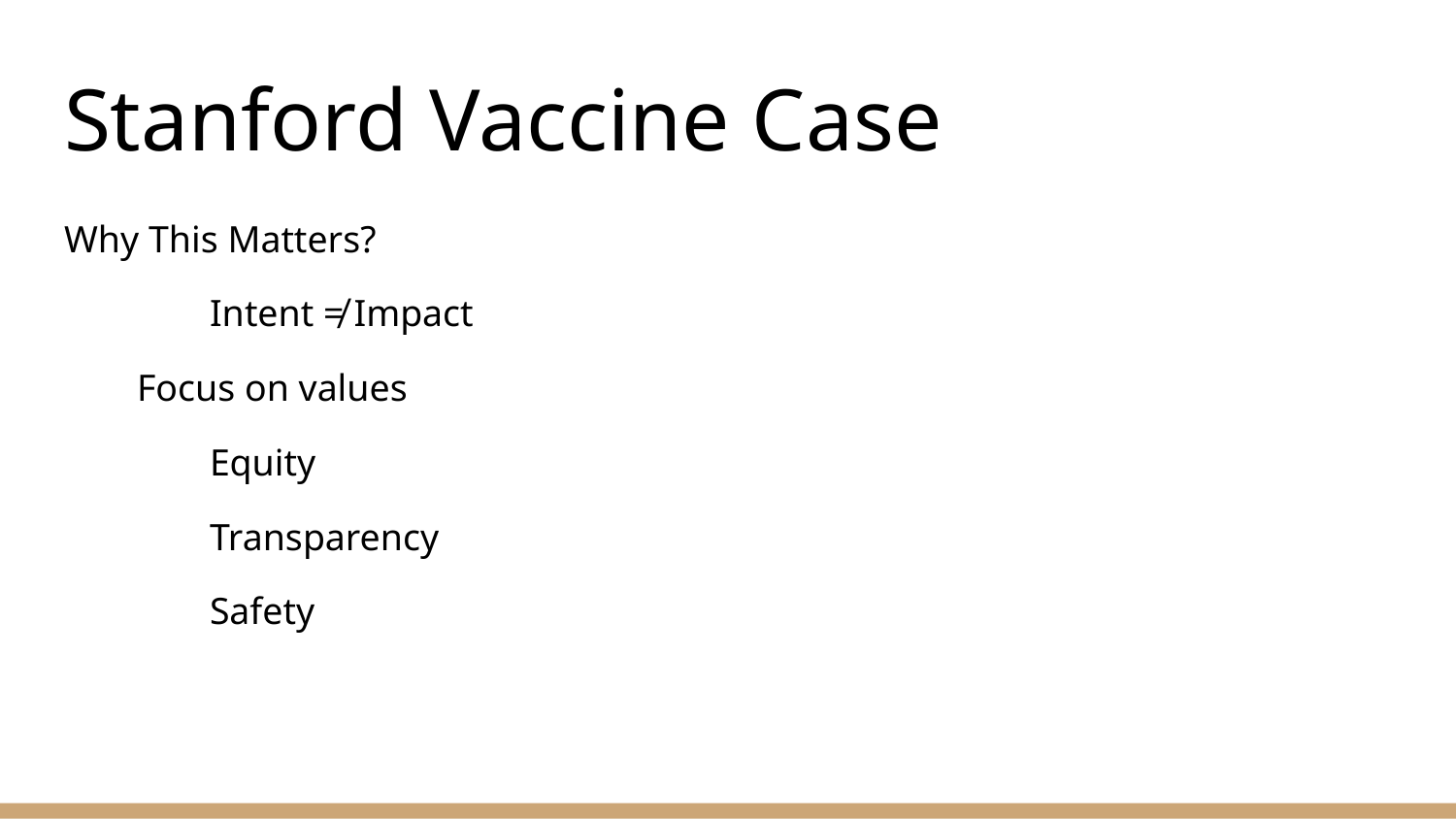

# Stanford Vaccine Case
Why This Matters?
	Intent ≠ Impact
Focus on values
Equity
Transparency
Safety

## Slide 21
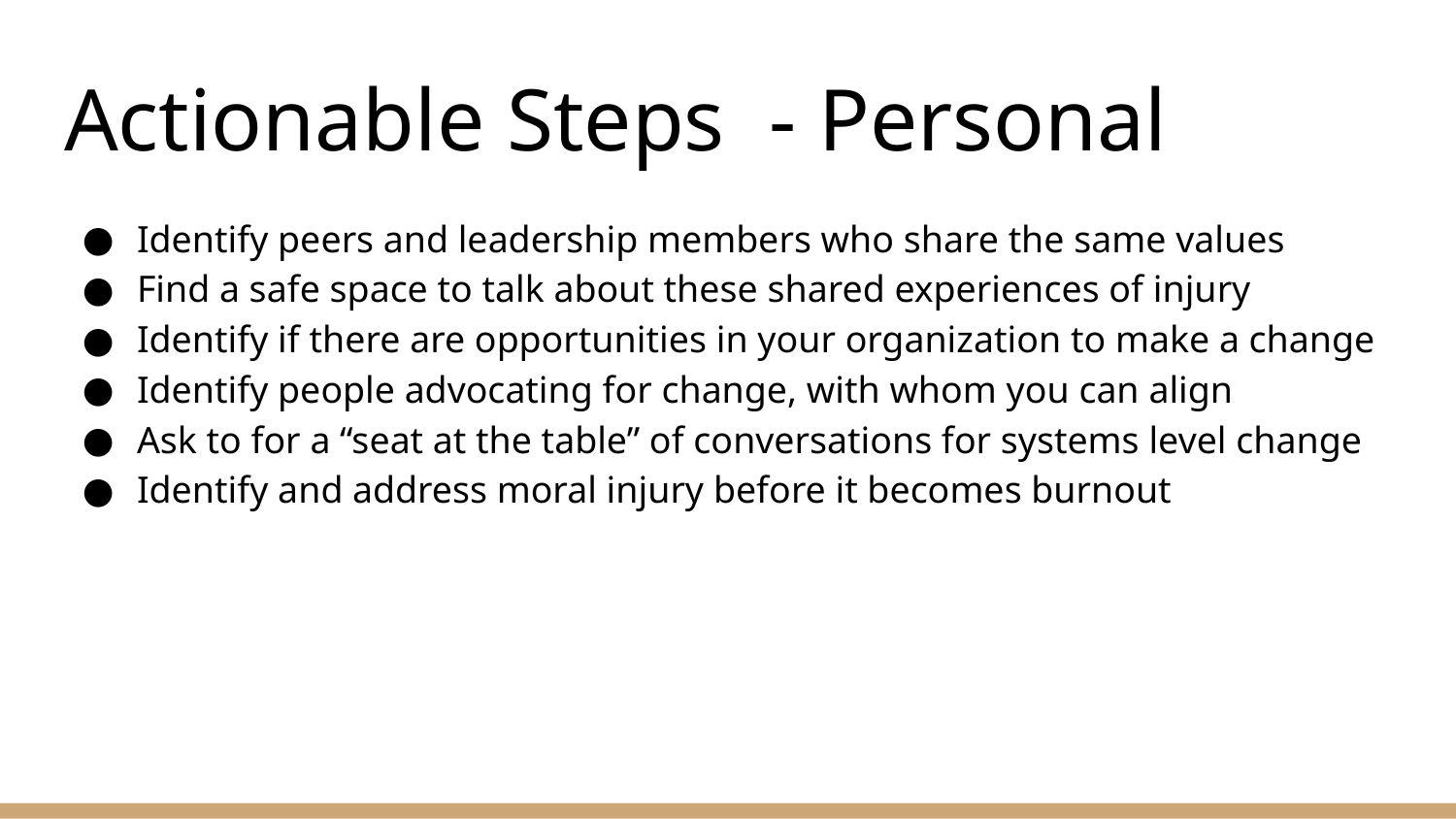

# Actionable Steps - Personal
Identify peers and leadership members who share the same values
Find a safe space to talk about these shared experiences of injury
Identify if there are opportunities in your organization to make a change
Identify people advocating for change, with whom you can align
Ask to for a “seat at the table” of conversations for systems level change
Identify and address moral injury before it becomes burnout

## Slide 22
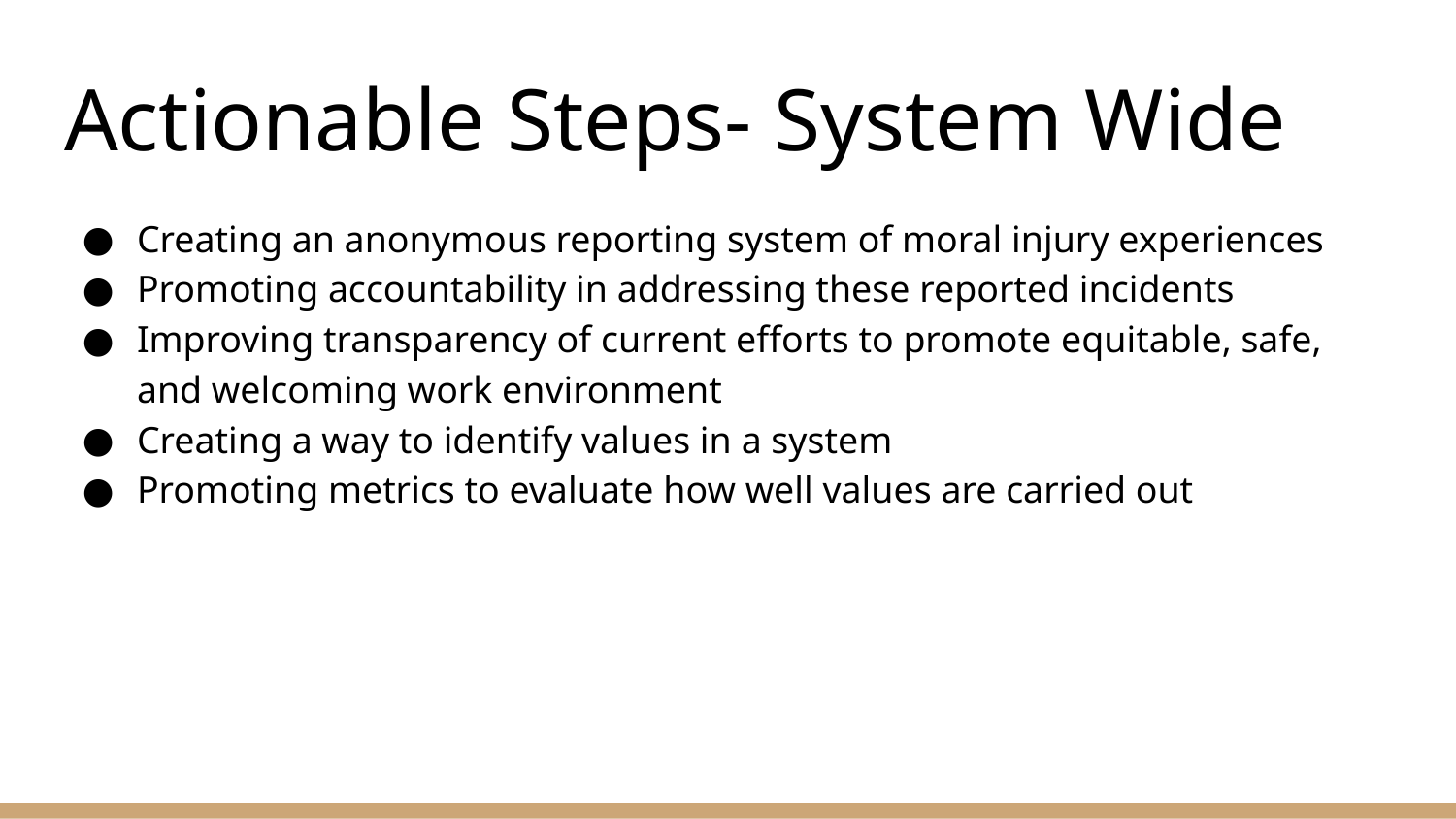

# Actionable Steps- System Wide
Creating an anonymous reporting system of moral injury experiences
Promoting accountability in addressing these reported incidents
Improving transparency of current efforts to promote equitable, safe, and welcoming work environment
Creating a way to identify values in a system
Promoting metrics to evaluate how well values are carried out

## Slide 23
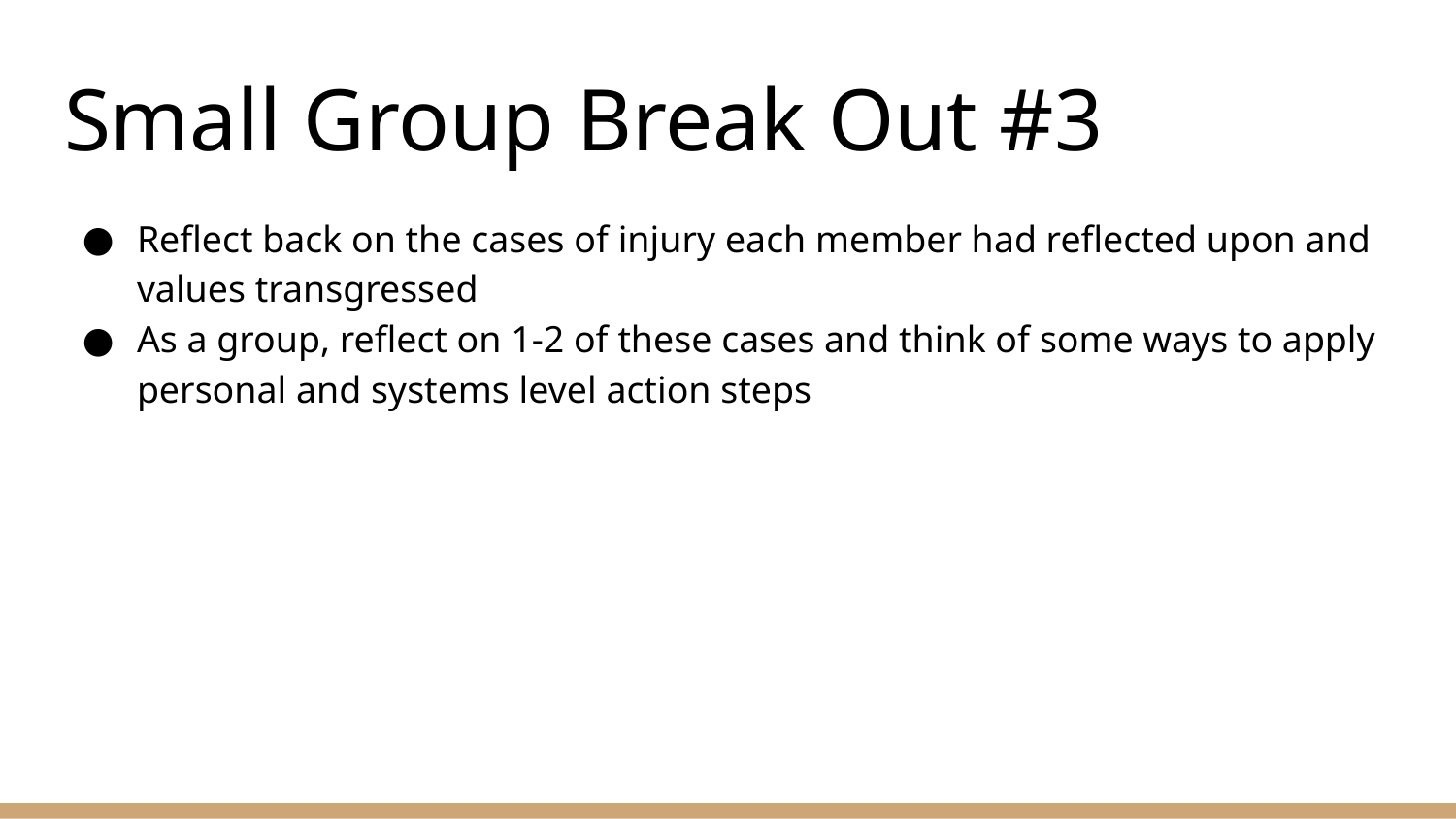

# Small Group Break Out #3
Reflect back on the cases of injury each member had reflected upon and values transgressed
As a group, reflect on 1-2 of these cases and think of some ways to apply personal and systems level action steps

## Slide 24
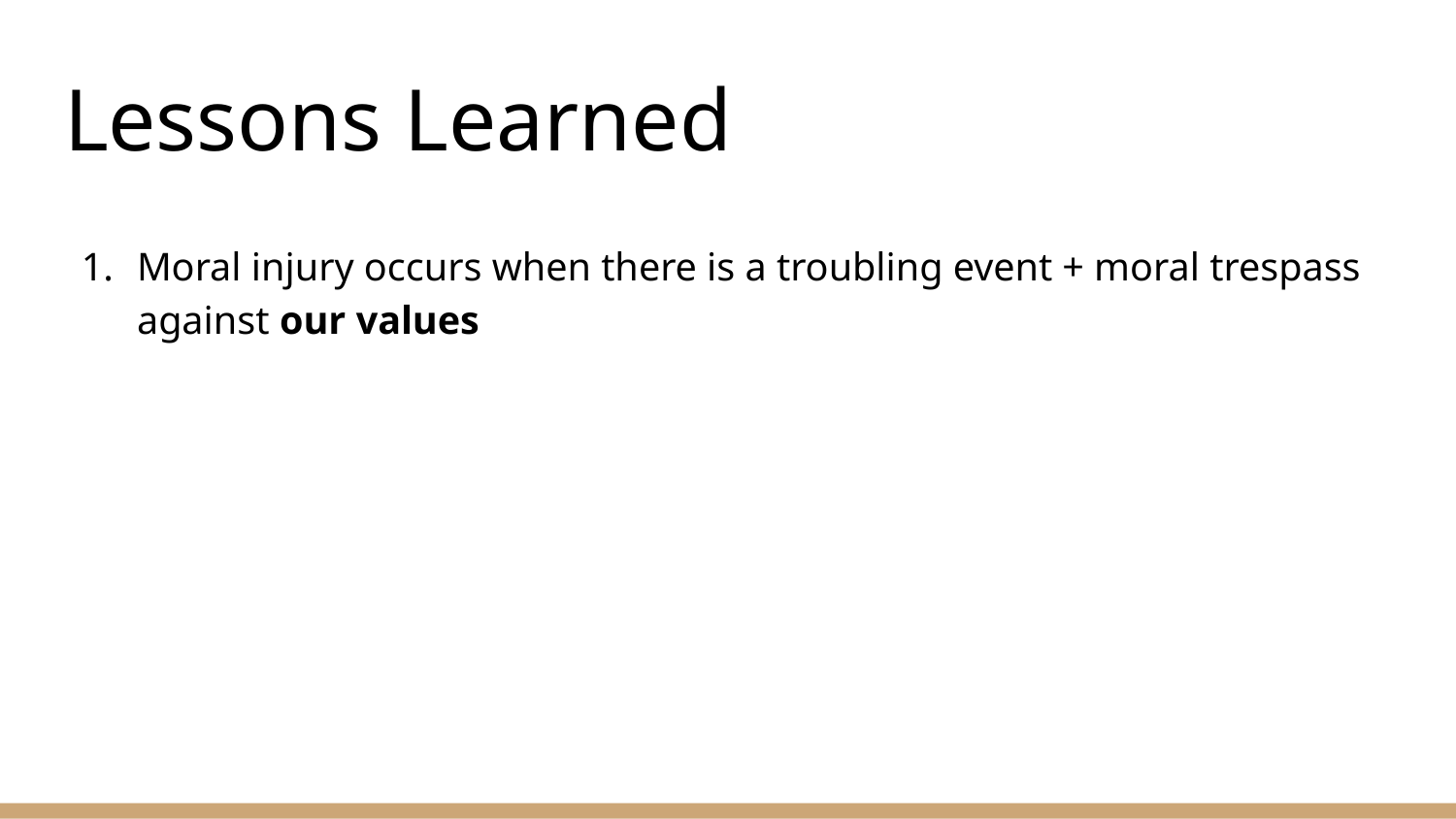

# Lessons Learned
Moral injury occurs when there is a troubling event + moral trespass against our values

## Slide 25
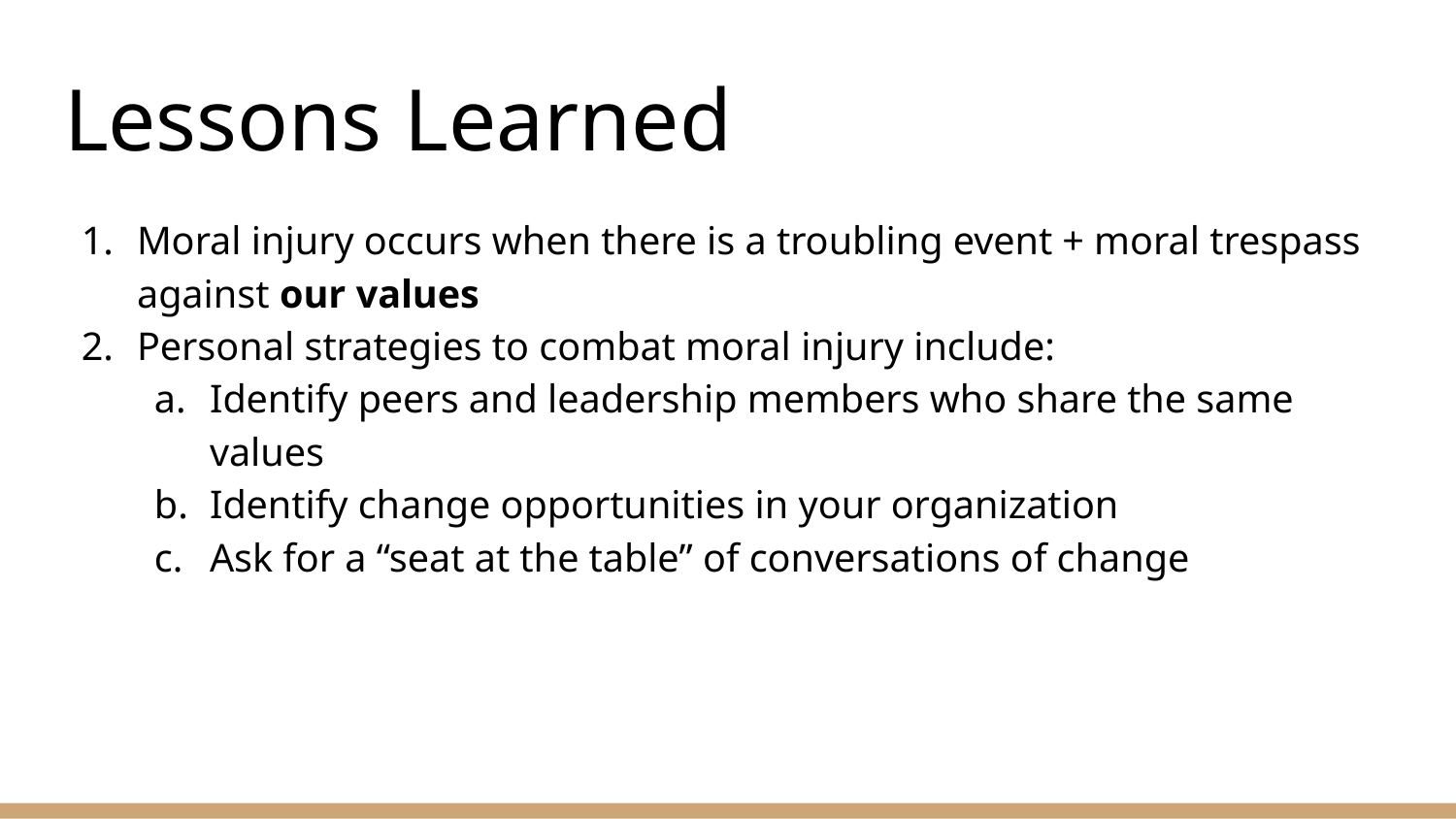

# Lessons Learned
Moral injury occurs when there is a troubling event + moral trespass against our values
Personal strategies to combat moral injury include:
Identify peers and leadership members who share the same values
Identify change opportunities in your organization
Ask for a “seat at the table” of conversations of change

## Slide 26
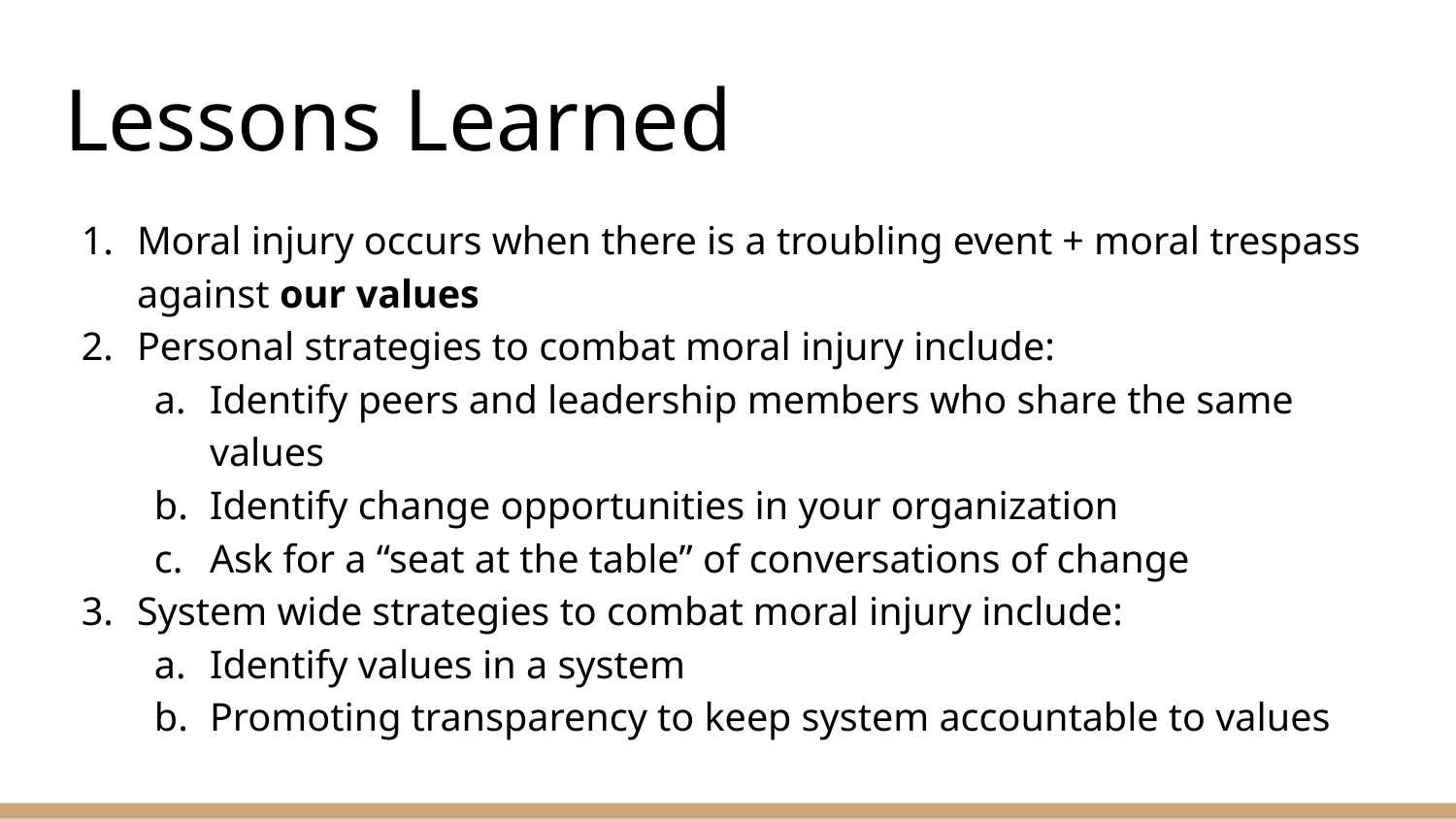

# Lessons Learned
Moral injury occurs when there is a troubling event + moral trespass against our values
Personal strategies to combat moral injury include:
Identify peers and leadership members who share the same values
Identify change opportunities in your organization
Ask for a “seat at the table” of conversations of change
System wide strategies to combat moral injury include:
Identify values in a system
Promoting transparency to keep system accountable to values

## Slide 27
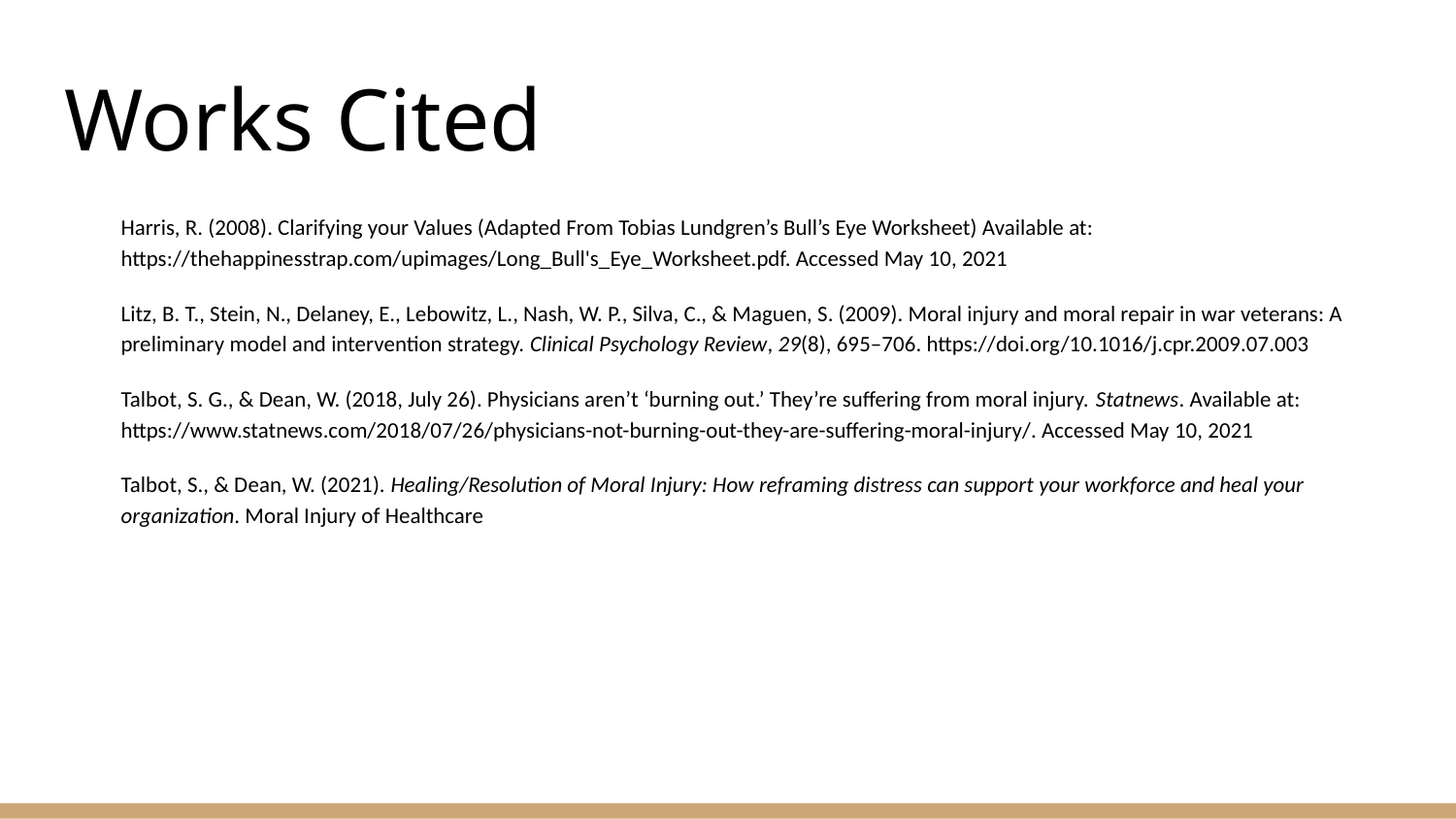

# Works Cited
Harris, R. (2008). Clarifying your Values (Adapted From Tobias Lundgren’s Bull’s Eye Worksheet) Available at: https://thehappinesstrap.com/upimages/Long_Bull's_Eye_Worksheet.pdf. Accessed May 10, 2021
Litz, B. T., Stein, N., Delaney, E., Lebowitz, L., Nash, W. P., Silva, C., & Maguen, S. (2009). Moral injury and moral repair in war veterans: A preliminary model and intervention strategy. Clinical Psychology Review, 29(8), 695–706. https://doi.org/10.1016/j.cpr.2009.07.003
Talbot, S. G., & Dean, W. (2018, July 26). Physicians aren’t ‘burning out.’ They’re suffering from moral injury. Statnews. Available at: https://www.statnews.com/2018/07/26/physicians-not-burning-out-they-are-suffering-moral-injury/. Accessed May 10, 2021
Talbot, S., & Dean, W. (2021). Healing/Resolution of Moral Injury: How reframing distress can support your workforce and heal your organization. Moral Injury of Healthcare

## Slide 28
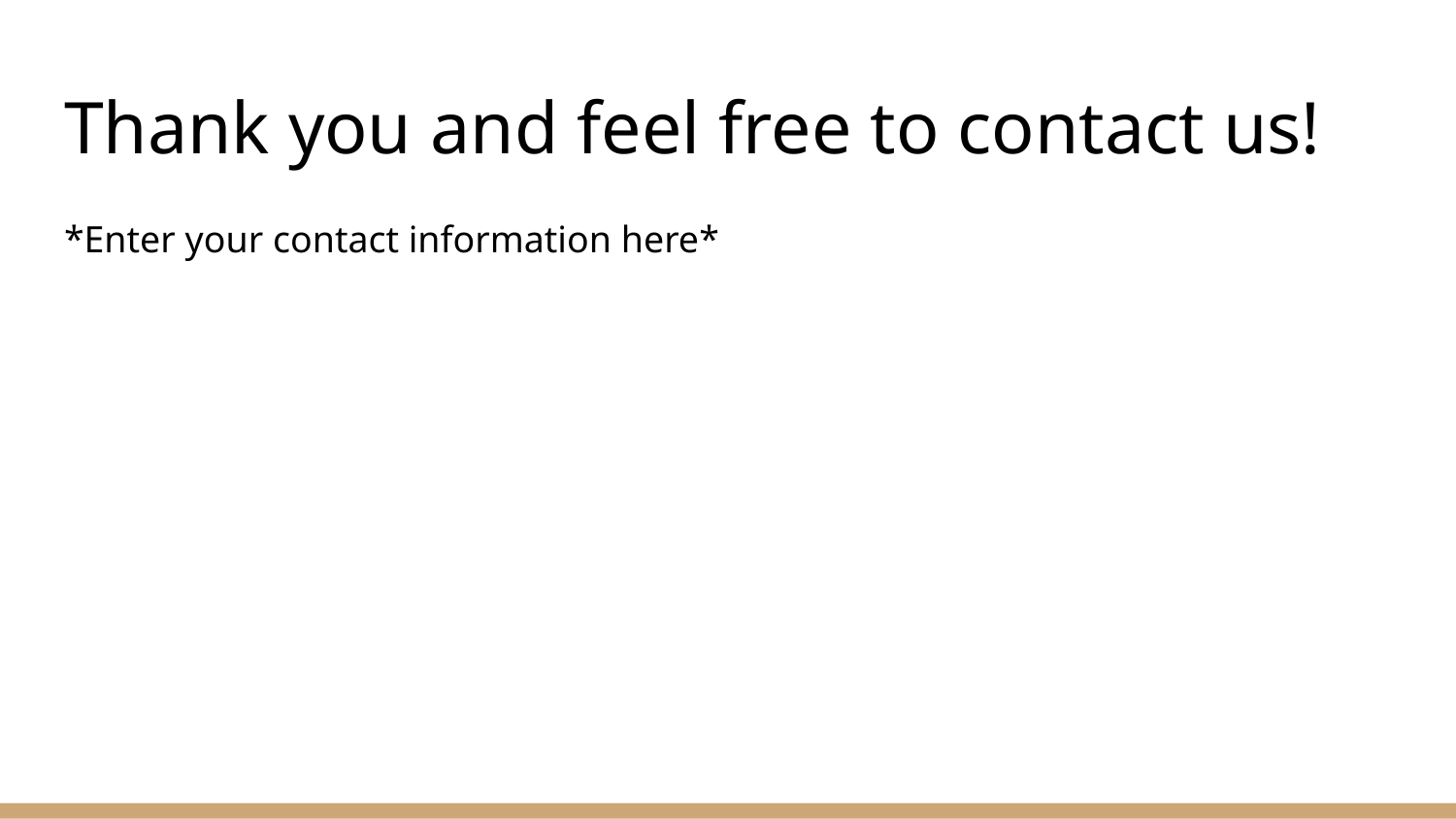

# Thank you and feel free to contact us!
*Enter your contact information here*
