# Supplementary material for: Moral Injury: How It Affects Us and Tools to Combat It
Source: MedEdPORTAL. 2023 Nov 3;19:11357. doi: 10.15766/mep_2374-8265.11357 (PMC10622333; doi:10.15766/mep_2374-8265.11357)
Supplement: Supplementary file 1 — Workshop Timeline.docxWorkshop Handout.docxWorkshop Evaluation.docxWorkshop PowerPoint.pptxFacilitator Guide.docxParticipant Takeaways.docx [file mep_2374-8265.11357-s001.zip › E. Facilitator Guide.docx]

**Title:** Moral Injury: How it affects us and tools to combat It

**Authors:** Connor Arquette, MD, Valerie Peicher, MD, Antonette Ajayi, MD, MPH, Dora Alvarez, MD, Alice Mao, MD, Tram Nguyen, MD, Anthony Sawyer, MD, MPH, Connie Martin Sears, MD, Eugene J. Carragee, MD, Baraka Floyd, MD, MSc, Bernadett Mahanay, C-TAGME, BA, Rebecca Blankenburg, MD, MPH

**Educational Objectives:**

By the end of this workshop, learners will be able to:

1. Define moral injury and relevant vocabulary
2. Recognize instances of moral injury and its effects on those around us
3. Develop strategies to combat moral injury

**Instructions for Small Group Case 1**

“In small groups, discuss this scenario and answer the following questions:

1) Has something like this ever happened to you and how did it make you feel?

2) How can we address the situation?

Please choose 1 team member to share your thoughts.”

Reiterate with group that moral injury comprises two components:

1. Troubling event
   1. In this case 1 example, the troubling event is the special treatment of the VIP patient
2. Moral trespass
   1. The values that may be violated in this example might include professionalism, justice, and equity but the group should feel free to provide their own along with their rationales

In order for an event to constitute as moral injury, it needs to transgress morals important to us as individuals or as a group. Refer and relate back to the bulls-eye worksheet where participants determined their values and how center they were to their core.

**Instructions for Large Group Reflections**

“All together, let’s talk about what each group discussed. Have you experienced something like this? How can we prevent future incidents?”

**Instructions for Small Group Case 2**

“In small groups, discuss this scenario and answer the following questions:

1) Has something like this ever happened to you and how did it make you feel?

2) How can we address the situation?

Please choose 1 team member to share your thoughts.”

In this example, the small groups are asked to formulate their own example of moral injury after hearing multiple examples during the presentation by the facilitators such as the Stanford vaccine case and the VIP patient case.

- In identifying their own example, small groups should describe the troubling event in their scenario and highlight the values that were transgressed consistent with the two requirements for an event to qualify as moral injury

**Instructions for Small Group Case 3**

The goal of this session is to use the examples previously identified and begin to formulate strategies to combat those instances of moral injury. Focus on 1-2 examples that were identified previously.

Facilitators: if the group has struggled to identify examples, you may use the examples from Appendix B (the workshop handout) or you may use the Stanford Vaccine Case. You should focus on identifying personal strategies that can be developed and implemented and also systems strategies. This should mirror the topics identified in the power point presentation. If the groups are struggling to identify solutions, use the themes on slides 21 and 22 to prompt the participants.

**Instructions for Large Group Reflections**

“All together, let’s talk about what each group discussed. Have you experienced something like this? How can we prevent future incidents?”

**Instructions for the Evaluation**

“Your feedback will help us improve our workshop. Please fill out the anonymous evaluation.”

**Frequently Asked Questions**

1. Does this constitute moral injury?
   1. As long as there is a troubling event and moral trespass, the two components for moral injury are satisfied.
2. Is there a limit to the number of values that can apply to a scenario?
   1. There is no limit. Often, situations will have multiple values that were violated.
3. Should the priority be on personal or systems level strategies to mitigate moral injury?
   1. The answer to this lies in what values were transgressed and whether they were personal values or values of the team/organization.
