# Supplementary material for: Moral Injury: How It Affects Us and Tools to Combat It
Source: MedEdPORTAL. 2023 Nov 3;19:11357. doi: 10.15766/mep_2374-8265.11357 (PMC10622333; doi:10.15766/mep_2374-8265.11357)
Supplement: Supplementary file 1 — Workshop Timeline.docxWorkshop Handout.docxWorkshop Evaluation.docxWorkshop PowerPoint.pptxFacilitator Guide.docxParticipant Takeaways.docx [file mep_2374-8265.11357-s001.zip › F. Participant Takeaways.docx]

Appendix F – Participant Takeaways

Lessons Learned

1. Moral injury occurs when there is a troubling event and moral trespass against our values
2. Personal strategies to combat moral injury include:
   1. Identify peers and leadership members who share the same values
      1. Finding those with similar values can help you more easily identify instances of moral injury
      2. Having a support system can help mitigate the effects of moral trespass when they do occur
   2. Identify change opportunities in your organization
   3. Ask for a “seat at the table” of conversation of change
      1. By joining the conversation, you can teach others about the importance of moral injury, help leaders identify instances of moral injury, and develop/share ways to mitigate and combat it
3. System wide strategies to combat moral injury include:
   1. Identify values in a system
      1. Understanding how the values of the system may differ from those of individuals can help determine how strategies at a system level may differ from those at a personal level
   2. Promoting transparency to keep system accountable to those values
      1. With reference to the Stanford vaccine case, transparency was one of the values transgressed and by leaders communicating the vaccine allotment determination process, the moral trespass and thus moral injury was mitigated
